# Clinical Outcomes and Postprocedural Antithrombotic Management After Left Atrial Appendage Occlusion in Patients with Gastrointestinal Bleeding: A Systematic Review and Expert-Informed Clinical Framework

**DOI:** 10.3390/biomedicines14081801

**Published:** 2026-08-11

**Authors:** Jonatan Vuković, Tina Bečić, Josipa Radić, Mislav Radić, Ljiljana Marčić, Damir Fabijanić, Ivana Jukić

**Affiliations:** 1 Department of Internal Medicine, Division of Gastroenterology, University Hospital of Split, 21000 Split, Croatia; jonatan.vukovic@mefst.hr; 2 Department of Internal Medicine, School of Medicine, University of Split, 21000 Split, Croatia; josiparadic1973@gmail.com (J.R.); mislavradic@gmail.com (M.R.); 3 Department of Cardiovascular Diseases, University Hospital of Split, 21000 Split, Croatia; tina.becic@gmail.com (T.B.); damirfabijanic62@gmail.com (D.F.); 4 Department of Internal Medicine, Division of Nephrology, Dialysis and Arterial Hypertension, University Hospital of Split, 21000 Split, Croatia; 5 Department of Internal Medicine, Division of Rheumatology, Allergology and Clinical Immunology, University Hospital of Split, 21000 Split, Croatia; 6 Department of Diagnostic and Interventional Radiology, University Hospital of Split, 21000 Split, Croatia; ljiljana.marcic@mefst.hr; 7 Department of Clinical Propedeutics, School of Medicine, University of Split, 21000 Split, Croatia; 8 Faculty of Health Sciences, University of Split, 21000 Split, Croatia

**Keywords:** left atrial appendage occlusion, atrial fibrillation, gastrointestinal bleeding, antithrombotic therapy, stroke prevention, device-related thrombosis, high bleeding risk, percutaneous interventions, cardiovascular outcomes, personalized medicine

## Abstract

**Background:** Patients with atrial fibrillation (AF) and a history of gastrointestinal (GI) bleeding represent a particularly challenging clinical population due to the coexistence of elevated thromboembolic and hemorrhagic risks. Percutaneous left atrial appendage occlusion (LAAO) has emerged as an alternative strategy for stroke prevention in patients in whom long-term oral anticoagulation is contraindicated or poorly tolerated. However, evidence specifically addressing clinical outcomes and optimal postprocedural antithrombotic management in this subgroup remains limited. **Methods:** A comprehensive systematic literature search was conducted across PubMed, Scopus, Web of Science, and Cochrane CENTRAL from database inception to April 2026. Studies were eligible if they included patients with prior gastrointestinal bleeding as the primary study population or reported separately extractable outcomes for a predefined gastrointestinal bleeding subgroup. The primary outcomes were recurrent GI bleeding, thromboembolic events, and all-cause mortality, while secondary outcomes included procedural success, device-related thrombosis, and postprocedural antithrombotic strategies. Owing to substantial clinical and methodological heterogeneity, findings were synthesized qualitatively in accordance with PRISMA 2020 recommendations. **Results:** Five observational studies reporting GI bleeding-specific outcomes were included in the systematic evidence synthesis. Procedural success rates were consistently high, and LAAO was associated with acceptable thromboembolic outcomes during follow-up. Nevertheless, recurrent GI bleeding remained a clinically relevant complication, particularly in patients with a prior bleeding history. Postprocedural antithrombotic regimens varied widely, ranging from short-term oral anticoagulation to dual or single antiplatelet therapy and reduced-intensity strategies. Less intensive regimens appeared feasible in carefully selected patients at very high bleeding risk; however, no universally optimal approach could be identified. **Conclusions:** Available observational evidence suggests that LAAO may represent a stroke prevention option in selected patients with AF and prior GI bleeding, although firm conclusions regarding net clinical benefit and the optimal postprocedural antithrombotic strategy cannot currently be drawn. Its net clinical benefit is closely linked to individualized postprocedural management, particularly the choice and intensity of antithrombotic therapy. These findings highlight the importance of a multidisciplinary, patient-centered approach and underscore the need for prospective studies to establish evidence-based treatment strategies in this high-risk population.

## 1. Introduction

Atrial fibrillation (AF) is the most common sustained cardiac arrhythmia and a major risk factor for ischemic stroke and systemic embolism [1,2]. Oral anticoagulation (OAC) remains the standard strategy for stroke prevention in patients with AF; however, its long-term use is frequently complicated by bleeding, particularly from the gastrointestinal (GI) tract. Among all major bleeding phenotypes encountered in anticoagulated patients, GI bleeding poses a distinct clinical challenge because it is common, often recurrent and frequently leads to interruption, modification or permanent discontinuation of antithrombotic therapy, thereby leaving patients insufficiently protected from cardioembolic events [3,4,5].

Because thrombus formation in non-valvular AF originates predominantly within the left atrial appendage (LAA), this structure has become a central therapeutic target in contemporary stroke prevention strategies [6,7]. Over the past decade, percutaneous left atrial appendage occlusion (LAAO) has emerged as an established non-pharmacological alternative for selected patients in whom long-term OAC is contraindicated, poorly tolerated or associated with an unfavorable balance between thromboembolic and bleeding risk [8,9,10]. This strategy is supported by randomized and observational evidence demonstrating non-inferiority to anticoagulation in selected populations [11,12,13]. In current clinical practice, this approach has gained increasing importance in patients with previous major bleeding, contraindications to anticoagulation or a markedly elevated hemorrhagic risk that makes conventional long-term anticoagulant treatment difficult to sustain safely [8,9,10].

Patients with prior GI bleeding represent one of the most clinically relevant subgroups within this high-risk population. They are frequently referred for LAAO precisely because anticoagulant therapy is often interrupted, dose-reduced or permanently discontinued after a hemorrhagic event, despite persistent thromboembolic risk. At the same time, previous GI bleeding is not a uniform clinical entity. Its clinical significance may differ according to bleeding source, severity, recurrence pattern, transfusion requirement, endoscopic findings and the presence of a definitively treated or potentially reversible lesion. These distinctions are likely to have important implications not only for candidacy for LAAO, but also for postprocedural management and long-term outcomes.

Although the role of LAAO has expanded considerably in contemporary practice, evidence specifically focused on patients with previous GI bleeding remains limited. Available observational data suggest that LAAO may be feasible in this population, with acceptable procedural safety and potentially favorable long-term outcomes, including in patients with a history of major GI bleeding [14,15,16]. At the same time, emerging evidence indicates that a history of GI bleeding may carry important prognostic implications even after successful device implantation and may identify patients at increased risk for subsequent bleeding events during follow-up [15,16,17]. Thus, in this subgroup, procedural success alone may not fully capture the true clinical effectiveness of LAAO.

Recent systematic evidence has emphasized that LAAO should be interpreted within a broader patient-centered clinical framework, particularly in vulnerable populations in whom procedural success alone may not fully reflect long-term clinical benefit [18]. This concept is particularly relevant in patients with prior GI bleeding, in whom postprocedural management may be as clinically important as the implantation procedure itself.

One of the most important unresolved issues in this setting is postprocedural antithrombotic therapy. Although temporary antithrombotic treatment after LAAO is generally required to reduce the risk of device-related thrombosis (DRT) during the endothelialization phase, the safest and most effective regimen in patients with previous GI bleeding has not been established. In current clinical practice, postimplant strategies vary substantially and may include short-term OAC, dual antiplatelet therapy (DAPT), single antiplatelet therapy (SAPT), reduced-dose direct oral anticoagulants (DOACs) or highly individualized regimens tailored to bleeding severity and thrombotic risk. Recent reviews and consensus-oriented publications have emphasized that post-LAAO antithrombotic management remains highly variable, particularly in patients at very high bleeding risk, and that robust evidence supporting one uniform strategy is lacking [19,20,21,22,23,24].

This therapeutic uncertainty has major clinical relevance. In patients with prior GI bleeding, an overly intensive postprocedural regimen may precipitate recurrent hemorrhage and diminish the practical benefit of LAAO, whereas an insufficiently protective regimen may increase the risk of DRT or embolic complications [25,26]. As a result, management after LAAO in this population requires more than technical procedural success; it requires careful integration of cardiovascular and gastroenterological risk assessment, detailed understanding of the prior bleeding episode and thoughtful tailoring of postprocedural therapy. The clinical significance of this issue is further underscored by the findings of Kikuchi et al., who demonstrated that previous GI bleeding is a relevant clinical marker in patients undergoing left atrial appendage closure and should not be treated as a marginal comorbidity in procedural decision-making [16].

Despite the growing recognition of this problem, no systematic review to date has comprehensively synthesized the available evidence on clinical outcomes after LAAO in patients with prior GI bleeding while simultaneously addressing postprocedural antithrombotic management as a central therapeutic challenge. Most published studies have either evaluated broader high-bleeding-risk cohorts or have reported GI bleeding only as one of several baseline clinical characteristics, without detailed analysis of its prognostic and therapeutic implications. Consequently, an important gap remains between expanding clinical use of LAAO in this population and the limited evidence available to guide postprocedural treatment decisions.

Therefore, the aim of this systematic review was to synthesize the available GI bleeding-specific evidence on clinical outcomes following percutaneous left atrial appendage occlusion in patients with a history of GI bleeding, with particular emphasis on postprocedural antithrombotic strategies. In addition, broader LAAO literature was considered separately in the Discussion to provide contextual support for the interpretation of postprocedural management.

## 2. Materials and Methods

### 2.1. Study Design and Registration

This systematic review was conducted in accordance with the Preferred Reporting Items for Systematic Reviews and Meta-Analyses (PRISMA 2020) guidelines and was prospectively registered in the PROSPERO database (registration number: CRD420261381985). The review was designed to synthesize the available evidence on the safety and clinical outcomes of percutaneous LAAO in patients with a history of GI bleeding, with particular emphasis on postprocedural antithrombotic strategies and their relationship to recurrent bleeding and thromboembolic events. Given the anticipated heterogeneity in study design, patient selection, definitions of GI bleeding and postprocedural antithrombotic regimens, a qualitative narrative synthesis was planned. The study protocol was developed prior to data extraction and the study selection process was structured according to predefined eligibility criteria.

### 2.2. Search Strategy

A comprehensive literature search was performed across four electronic databases: PubMed, Scopus, Web of Science, and Cochrane CENTRAL. The search covered all records from database inception to 27 April 2026. No language restrictions were applied during the initial search. The search strategy combined controlled vocabulary terms and free-text keywords related to LAAO, AF, GI bleeding and antithrombotic therapy.

The core search strategy included combinations of the following terms: (“left atrial appendage occlusion” OR “left atrial appendage closure” OR LAAO OR LAAC OR Watchman OR Amulet OR LAmbre) AND (“gastrointestinal bleeding” OR “GI bleeding” OR “gastrointestinal hemorrhage” OR “bleeding history”) AND (“atrial fibrillation” OR AF) AND (antithrombotic OR anticoagulation OR antiplatelet OR “postprocedural therapy” OR “device-related thrombosis”). The search strategy was adapted as required for the syntax of each individual database. To ensure completeness, the reference lists of all included studies and relevant review articles were also manually screened for additional eligible publications. The full database-specific search strategies, including exact Boolean operators, search fields, dates of search and database-specific adaptations, are provided in Supplementary Table S1. In addition, the number of records retrieved from each database was documented to improve the transparency, reproducibility and auditability of the search process. GI bleeding-specific subgroup data were identified through title and abstract screening, full-text assessment of studies including patients with previous bleeding and manual review of baseline characteristics, subgroup analyses and Supplementary Materials.

Because some relevant studies reported prior GI bleeding only as a subgroup characteristic or baseline bleeding phenotype rather than as a primary focus of the title, abstract, or indexing terms, the search process was complemented by manual screening of reference lists and full-text evaluation of broader high-bleeding-risk LAAO studies for potentially relevant GI bleeding-specific data. The systematic search was designed to identify studies reporting clinical outcomes in patients with prior GI bleeding, either as the primary study population or as an analyzable subgroup. Broader high-bleeding-risk LAAO studies without extractable GI bleeding-specific outcomes were not eligible for inclusion in the systematic evidence synthesis. Relevant broader studies were considered separately as contextual literature in the Discussion and were not included in the PRISMA flow, study-level evidence tables or formal risk-of-bias synthesis.

### 2.3. Eligibility Criteria

Studies were considered eligible if they met the following criteria: (1) included adult patients with AF undergoing percutaneous LAAO; (2) reported data on patients with a history of GI bleeding, either as the primary study population or as a predefined subgroup; (3) provided information on at least one clinically relevant outcome, including procedural success, recurrent GI bleeding, thromboembolic events, mortality, DRT, or postprocedural antithrombotic therapy; and (4) were original peer-reviewed studies, including prospective or retrospective cohort studies, registry analyses and observational studies.

Studies were excluded if they: (1) involved surgical or thoracoscopic LAA exclusion rather than percutaneous occlusion; (2) did not report outcomes separately for patients with prior GI bleeding when mixed populations were included; (3) were case reports, small case series, conference abstracts, editorials, letters, narrative reviews, or expert opinion papers; and (4) lacked sufficient clinical detail relevant to the objectives of this review.

For the systematic evidence synthesis, eligible studies were required either to enroll patients with prior GI bleeding as the primary study population or to provide separately extractable outcome data for a predefined GI bleeding subgroup. Broader high-bleeding-risk LAAO studies without separately extractable GI bleeding-specific outcomes were excluded from the formal systematic evidence base. Such studies could be cited separately in the Discussion when relevant to the interpretation of postprocedural antithrombotic management, but they were not included in the PRISMA flow, study-level evidence tables or formal risk-of-bias assessment.

Reporting of postprocedural antithrombotic therapy was not mandatory for inclusion if at least one other predefined clinically relevant outcome was available. Accordingly, lack of antithrombotic therapy data alone was not considered sufficient for exclusion unless the study also lacked other outcomes relevant to the objectives of this review.

### 2.4. Study Selection

**Figure 1:**
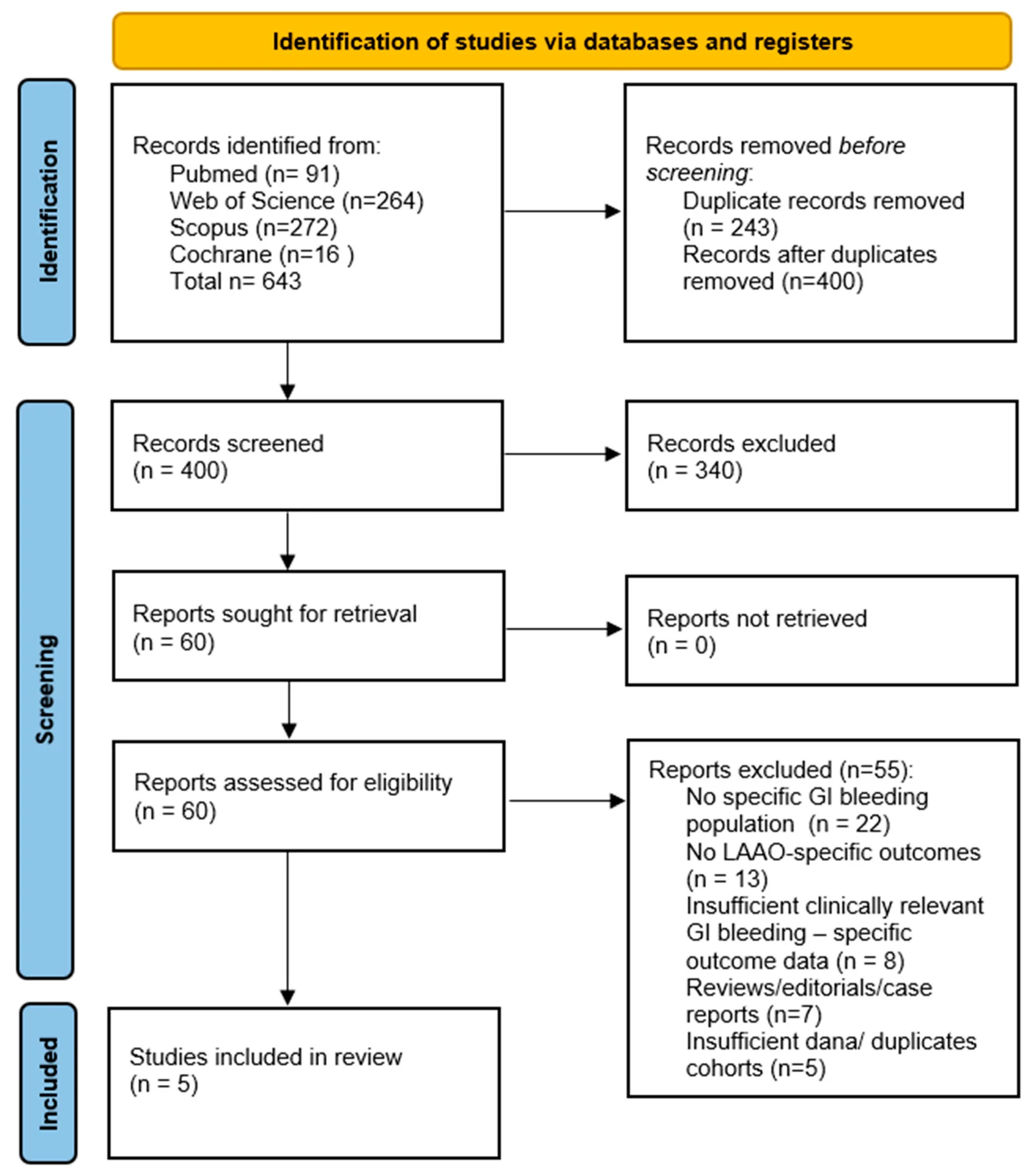
PRISMA 2020 flow diagram of study selection. A total of 643 records were identified through database searching. After removal of 243 duplicate records, 400 unique records underwent title and abstract screening, of which 340 were excluded as not relevant to the review question. The full texts of 60 articles were assessed for eligibility and 55 were excluded according to the predefined inclusion and exclusion criteria. Ultimately, five studies reporting GI bleeding-specific outcomes were included in the qualitative systematic synthesis.

All records identified through the database search were imported into a reference management system (Zotero, version 9.0.6; Corporation for Digital Scholarship, Vienna, VA, USA), and duplicates were removed. Two reviewers independently screened titles and abstracts for potential eligibility. Full-text articles deemed potentially relevant were subsequently assessed independently according to the predefined inclusion and exclusion criteria. Any disagreements between reviewers were resolved through discussion and consensus. If necessary, a third reviewer was consulted to resolve persistent discrepancies. The entire study selection process was documented and is summarized in Figure 1 using a PRISMA flow diagram. A detailed list of full-text articles excluded after eligibility assessment, together with the specific reason for exclusion, is provided in Supplementary Table S2. 

### 2.5. Data Extraction

Data extraction was performed independently by two reviewers (JV and TB) using a standardized data extraction form developed specifically for this review. The following variables were extracted from each included study: first author, year of publication, country, study design, sample size, baseline patient characteristics, definition and history of gastrointestinal bleeding, type of LAAO device, procedural success, periprocedural complications, postprocedural antithrombotic regimen, duration of follow-up, recurrent GI bleeding, thromboembolic events, DRT and mortality.

Whenever available, additional data were collected regarding the source of GI bleeding, timing of previous bleeding episodes in relation to the LAAO procedure, endoscopic findings, therapeutic interventions for the bleeding source and any modifications of antithrombotic therapy during follow-up. Any discrepancies in extracted data were resolved by consensus between the reviewers. Where available, these variables were summarized in the study characteristics and supplementary evidence tables, specifically Supplementary Table S3. When study-level reporting was incomplete or inconsistent, this was explicitly acknowledged in the narrative synthesis.

### 2.6. Outcomes of Interest

The primary outcomes of interest were clinical outcomes following percutaneous LAAO in patients with a history of gastrointestinal bleeding, including recurrent GI bleeding, thromboembolic events and all-cause mortality during follow-up.

Secondary outcomes included procedural success, periprocedural complications, DRT, peri-device leak when reported and the type and duration of postprocedural antithrombotic therapy. Particular attention was given to the variability of postprocedural antithrombotic regimens and to potential associations between treatment strategy and subsequent bleeding or thromboembolic outcomes. Because the included studies used heterogeneous definitions for recurrent GI bleeding, major bleeding, thromboembolic events, procedural success and DRT, no formal harmonization of outcome definitions was performed. Instead, outcomes were extracted and synthesized according to the definitions used in the original studies and are therefore presented narratively.

### 2.7. Methodological Quality and Risk-of-Bias Assessment

The methodological quality and risk of bias of the included observational studies were assessed using the Joanna Briggs Institute Critical Appraisal Checklist for Cohort Studies. This tool was selected because the eligible evidence base consisted of observational cohort studies, registry analyses and a propensity-matched cohort rather than randomized trials or uniformly comparative non-randomized intervention studies.

The assessment considered the similarity and recruitment of comparison groups, measurement of exposure, identification and management of confounding factors, baseline outcome status, validity and reliability of outcome measurement, adequacy and completeness of follow-up and appropriateness of statistical analysis. For studies without a formal comparison group, items requiring between-group comparison were recorded as not applicable and were not used to penalize the overall methodological assessment.

Two reviewers (JV and TB) independently performed the assessment. Disagreements were resolved through discussion and consensus. Individual item-level judgments and supporting explanations for each study are provided in Supplementary Table S4. Because the JBI checklist does not prescribe a mandatory numerical threshold or standardized overall risk-of-bias category, the appraisal was interpreted qualitatively rather than converted into an arbitrary summary score.

### 2.8. Data Synthesis

Because substantial heterogeneity was anticipated across study populations, definitions of gastrointestinal bleeding, LAAO devices, outcome measures and postprocedural antithrombotic regimens, a meta-analysis was not planned unless sufficient methodological and clinical homogeneity was identified. Accordingly, the findings were synthesized qualitatively using a narrative approach. Before undertaking the synthesis, the included studies were formally assessed for potential quantitative pooling according to population definition, outcome definition, comparator structure, follow-up duration and availability of compatible effect estimates. Meta-analysis was not performed because no predefined outcome was reported with sufficient clinical and methodological comparability across an adequate number of studies. The principal sources of clinical and methodological heterogeneity that precluded quantitative pooling are summarized in Supplementary Table S5.

The synthesis focused on three principal domains: (1) procedural and clinical outcomes of LAAO in patients with prior GI bleeding; (2) recurrence of bleeding and thromboembolic events during follow-up; (3) postprocedural antithrombotic management strategies, including their variability and potential clinical implications. To improve the structure and interpretability of the narrative synthesis, study-level outcome data were also summarized in a structured evidence table, including follow-up duration, recurrent GI bleeding, thromboembolic events or stroke/systemic embolism, DRT, mortality and postprocedural antithrombotic regimen, where available. Where feasible, findings were additionally interpreted according to bleeding source, antithrombotic regimen and study design. No formal meta-analysis was performed due to substantial clinical and methodological heterogeneity across the included studies. Assessment of publication bias was not performed due to the limited number and heterogeneity of included studies. A formal GRADE assessment of certainty of evidence was not performed because of the substantial clinical and methodological heterogeneity of the included studies, as well as the predominance of observational evidence.

## 3. Results

### 3.1. Study Selection

A total of 643 records were identified through the database search. Following removal of 243 duplicates, 400 unique records underwent title and abstract screening. Of these, 340 were excluded as not relevant to the review question. The full texts of 60 articles were assessed for eligibility, of which 55 were excluded according to the predefined inclusion and exclusion criteria. Ultimately, five studies were included in the qualitative systematic synthesis.

### 3.2. Study Characteristics

The five studies included in the systematic evidence synthesis comprised observational cohorts, registry analyses and one propensity-matched cohort, with their main characteristics summarized in Table 1.

Across the five study cohorts, the GI bleeding-specific denominators comprised 85,633 patients (151, 19, 358, 84,472, and 633, respectively). However, 84,472 patients (98.6%) were derived from a single registry, whereas the remaining four studies included only 1161 patients in total.

Of the five included studies, two specifically enrolled patients with previous gastrointestinal bleeding and three provided separately analyzable GI bleeding subgroup data. Broader high-bleeding-risk studies without extractable GI bleeding-specific outcomes were not included in the systematic evidence synthesis and are discussed separately only when relevant to contextual interpretation.

The included studies evaluated several LAAO devices, most commonly WATCHMAN and Amplatzer-based devices. Follow-up duration varied across studies, ranging from early postprocedural assessment to long-term follow-up. Outcomes of interest included procedural success, recurrent bleeding, thromboembolic events, DRT, mortality and postprocedural antithrombotic therapy. Where reported, additional study-level data were extracted regarding the source and severity of prior GI bleeding, timing of bleeding episodes in relation to LAAO, endoscopic evaluation or treatment of the bleeding source and modifications of antithrombotic therapy during follow-up. However, reporting of these variables was inconsistent across studies, which limited direct comparison and contributed to the narrative nature of the synthesis. Detailed GI-specific variables are summarized in Supplementary Table S3. A structured summary of study-level clinical outcomes and postprocedural antithrombotic regimens is provided in Table 2.

### 3.3. Clinical Outcomes After LAAO

Overall, the available evidence suggests that LAAO is a feasible stroke prevention strategy in patients with AF and a history of GI bleeding. Procedural success was generally high across the included studies, whereas rates of recurrent GI bleeding, thromboembolic events, DRT and mortality varied substantially according to study population, baseline bleeding history, follow-up duration and postprocedural antithrombotic regimen.

Studies specifically focusing on patients with previous GI bleeding suggest that this population represents a distinct high-risk subgroup. Although these patients may benefit from reduced long-term exposure to OAC, they remain vulnerable to recurrent bleeding, especially during the early postprocedural period when temporary antithrombotic therapy is still required. Follow-up duration varied substantially across the included studies. Exact study-level outcome data are summarized in Table 2 to facilitate comparison across heterogeneous study populations.

### 3.4. Postprocedural Antithrombotic Therapy

Postprocedural antithrombotic regimens varied substantially across the included studies. Reported strategies included short-term OAC, DAPT, SAPT, half-dose DOAC therapy and simplified regimens with reduced antithrombotic intensity.

The available evidence suggests that less intensive postprocedural regimens may be a reasonable option in carefully selected patients at very high bleeding risk. However, the data remain heterogeneous and no single regimen can currently be regarded as universally optimal. Accordingly, postprocedural antithrombotic therapy should be individualized on the basis of thromboembolic risk, the severity and recurrence pattern of prior bleeding, adequacy of control of the GI bleeding source and the risk of DRT. Study-level regimen variability and associated reported outcomes are summarized in Table 2.

### 3.5. Risk of Bias

#### Methodological Quality and Risk of Bias

The methodological quality of the five included observational studies was assessed using the JBI Critical Appraisal Checklist for Cohort Studies. The principal methodological limitations were related to residual confounding, differences in baseline clinical characteristics, incomplete reporting of GI bleeding phenotype and source control and variability in follow-up and outcome ascertainment. Several studies used registry-based or retrospective designs, which increased the possibility of selection bias and limited control of clinically relevant confounders.

Outcome measurement was generally based on clinical records, registry data or predefined study endpoints, although definitions of recurrent GI bleeding and other clinical outcomes were not uniform across studies. Follow-up was reported in all included studies, but the handling of incomplete follow-up and missing data was not consistently described. Overall, the evidence base was considered methodologically limited and the findings should therefore be interpreted cautiously. Detailed item-level assessments and supporting judgments are provided in Supplementary Table S4, while a concise study-level summary is presented in Table 3.

## 4. Discussion

This systematic review synthesizes the available evidence on clinical outcomes following percutaneous LAAO in patients with a history of GI bleeding, with particular emphasis on postprocedural antithrombotic strategies. The main findings of this review are threefold. First, available observational evidence suggests that LAAO may represent a feasible stroke prevention option in carefully selected patients with prior GI bleeding, although the certainty of this conclusion remains limited. Second, although recurrent GI bleeding after LAAO appears to be less frequent than might be expected in this high-risk population, it remains a clinically relevant event and is likely influenced not only by the procedural intervention itself, but also by the underlying bleeding substrate and the postprocedural antithrombotic regimen. Third, the available evidence demonstrates substantial heterogeneity in postprocedural antithrombotic management, underscoring the absence of a standardized therapeutic approach and highlighting one of the most important unresolved issues in the care of these patients [14,15,16,19,24,28,29]. Importantly, the randomized evidence included in this review was not derived from a GI bleeding-specific trial population. Randomized data were therefore interpreted cautiously and used primarily to contextualize broader LAAO management rather than to support direct conclusions for patients with prior GI bleeding. The interpretation of these findings should be considered in conjunction with the structured study-level outcome data presented in Table 3, which illustrate that procedural feasibility, recurrent bleeding, thromboembolic events and postprocedural antithrombotic strategies were reported heterogeneously across studies. Importantly, this review remains centered on GI bleeding-specific evidence. Broader high-bleeding-risk and randomized LAAO studies were incorporated only as contextual support and should not be interpreted as equivalent to the direct evidence base derived from studies specifically addressing prior GI bleeding. Accordingly, the discussion below distinguishes, wherever possible, between conclusions supported directly by GI bleeding-specific evidence and those informed more broadly by contextual high-bleeding-risk LAAO literature. Conclusions directly related to prior GIbleeding are derived primarily from GI bleeding-specific studies and analyzable GI bleeding subgroups rather than from broader randomized or high-bleeding-risk evidence. Importantly, much of the directly relevant evidence in this field remains observational, heterogeneous and at least moderately limited by risk of bias, whereas the available randomized evidence is indirect with respect to the specific question of prior GI bleeding. Accordingly, the interpretations presented below should be viewed as low-certainty and primarily hypothesis-generating rather than definitive.

Patients with AF and a history of GI bleeding represent a particularly challenging clinical population because they exist at the intersection of two competing risks: thromboembolism and recurrent hemorrhage. In routine practice, prior GI bleeding often leads to interruption, dose reduction, switching or complete discontinuation of OAC, thereby exposing patients to residual cardioembolic risk. This paradigm is well established in contemporary AF guidelines and observational studies addressing anticoagulation interruption [1,2,3,4,5,30,31]. In this context, LAAO provides an attractive non-pharmacological alternative by targeting the principal site of thrombus formation in non-valvular AF while potentially reducing dependence on long-term systemic anticoagulation. The findings synthesized in this review suggest that LAAO may offer a potentially favorable balance between ischemic protection and bleeding avoidance in this difficult population, although the available evidence remains largely observational, heterogeneous and subject to important methodological limitations, and should therefore be interpreted with appropriate caution [11,12,13,14,15,16,17,32,33,34].

A central point emerging from the current literature is that prior GI bleeding should not be viewed as a uniform clinical entity. Rather, it encompasses a broad range of bleeding phenotypes that differ substantially in pathophysiology, reversibility and prognostic significance. Patients with previously treated, clearly identifiable and potentially correctable bleeding lesions may differ markedly from those with diffuse angiodysplasia, portal hypertensive lesions, recurrent obscure bleeding or multiple coexisting sources of hemorrhage. This heterogeneity likely contributes to the variability in outcomes reported across studies and may partly explain why postprocedural management after LAAO remains highly individualized in this setting. It also suggests that patient selection for LAAO should not rely solely on the presence or absence of previous GI bleeding, but should incorporate a more detailed characterization of bleeding mechanism, recurrence pattern, and treatability. This interpretation is supported by more recent cohort data showing that patients with prior GI bleeding constitute a clinically distinct subgroup and may experience different bleeding trajectories after LAAO than other high-bleeding-risk populations [15,17]. This concept is further reinforced by recent contemporary evidence. Darden et al. demonstrated that prior GI bleeding identifies a clinically vulnerable subgroup of patients undergoing LAAO, characterized by higher rates of bleeding-related complications and mortality despite similar stroke prevention outcomes [17]. Preisendörfer et al. likewise showed that patients with AF and major GI bleeding remain at increased risk during follow-up even after successful LAAO [27]. Taken together, these studies strengthen the view that, in this population, procedural success should not be equated with resolution of long-term bleeding vulnerability and that the postprocedural phase remains a major determinant of overall clinical benefit [17,27]. The following broader LAAO studies were not included in the formal systematic evidence synthesis because they did not provide separately extractable GI bleeding-specific outcomes. They are discussed solely as contextual literature relevant to the interpretation of procedural outcomes and postprocedural antithrombotic management and should not be considered part of the direct evidence base of this review. The CLOSURE-AF trial failed to demonstrate noninferiority of LAAO compared with best medical therapy in older high-risk patients, underscoring that the clinical benefit of LAAO should not be overstated in fragile populations with competing bleeding and thromboembolic risks. Although CLOSURE-AF was not designed specifically for patients with prior GI bleeding, it provides important contextual evidence supporting a measured interpretation of benefit in patients with substantial baseline vulnerability. CLOSURE-AF was therefore interpreted as contextual randomized evidence rather than as part of the direct GI bleeding-specific evidence base.

One of the most clinically important findings of this review is the marked variability in postprocedural antithrombotic therapy. Although temporary antithrombotic treatment after LAAO is generally recommended to reduce the risk of DRT during the endothelialization phase, no consensus has been established regarding the optimal regimen for patients with previous GI bleeding. In the broader LAAO literature, postprocedural strategies have included short-term OAC, DAPT, SAPT, reduced-dose DOACs and combinations modified according to procedural findings or individual risk factors. These strategies have evolved significantly from early warfarin-based protocols toward more flexible regimens [11,12,24,35,36,37,38,39,40]. However, in patients with prior GI bleeding, the choice of therapy becomes especially complex because the protective benefit against DRT must be weighed against the possibility of precipitating recurrent hemorrhage. Contemporary reviews have consistently emphasized that post-LAAO antithrombotic management remains highly heterogeneous and is still driven more by clinical judgment than by direct comparative evidence, especially in patients at very high bleeding risk [20,21,22,23,25]. Recent randomized and comparative evidence has further refined this discussion. The ADALA trial suggested that a low-dose DOAC-based strategy may offer a favorable short-term balance compared with DAPT after LAAO [41]; however, its small sample size and premature termination limit the strength and generalizability of this signal. Likewise, the ANDES study suggested that short-term DOAC therapy may provide protection against DRT comparable to DAPT, while potentially reducing bleeding complications [42], although interpretation is limited by relatively short follow-up and open-label design. These randomized and comparative data are clinically relevant for contextual interpretation of post-LAAO antithrombotic strategies, but they do not constitute direct GI bleeding-specific evidence. In addition, a recent network meta-analysis ranked DOAC-based strategies, particularly low-dose DOAC regimens, favorably in terms of post-LAAO safety and efficacy, but the certainty of evidence remained low to very low [43]. Taken together, these data suggest that lower-intensity DOAC-based approaches may be promising in selected patients, but they should still be regarded as hypothesis-generating and should not be interpreted as establishing a definitive standard of care, particularly in patients with prior GI bleeding [41,42,43].

Despite these emerging data, this therapeutic uncertainty has important practical implications. From a mechanistic perspective, insufficient postprocedural antithrombotic therapy may increase the risk of DRT, which in turn may compromise the very objective of LAAO by allowing thromboembolic events to occur despite successful implantation. Conversely, overly intensive antithrombotic therapy may negate the bleeding-related advantage of the procedure, particularly in patients with fragile mucosal lesions or recurrent bleeding predisposition. The challenge, therefore, is not merely to perform LAAO successfully, but to identify the minimum effective antithrombotic strategy that preserves device safety without reintroducing the bleeding burden that initially led to consideration of the procedure. This balance is particularly difficult in the absence of randomized data specifically focused on patients with previous GI bleeding. The importance of this issue is reinforced by evidence linking postimplant antithrombotic choices to the prevention of device-related thrombosis, a complication that remains one of the most feared adverse events after LAAO [22,25,26,44].

The available evidence also suggests that clinical outcomes after LAAO in patients with prior GI bleeding are shaped by factors extending beyond cardiology alone. A multidisciplinary approach appears especially important in this population. Preprocedural gastroenterological evaluation may help identify the cause of prior bleeding, assess whether the lesion is definitively treated or remains at risk for recurrence and inform the expected tolerance of short-term antithrombotic therapy after implantation. Similarly, postprocedural management may benefit from coordinated follow-up involving both cardiology and gastroenterology, particularly in patients with recurrent anemia, occult bleeding or prior hospitalization for major hemorrhage. In this sense, successful LAAO in patients with GI bleeding should be understood not simply as a device-based intervention, but as part of a broader integrated management strategy. This broader framing is consistent with current reviews and guidance documents that emphasize individualized postimplant therapy and structured follow-up rather than a purely procedural definition of success [8,10,20,21,23,25]. However, these considerations should be interpreted as emerging clinical implications of the available evidence rather than as conclusions derived uniformly from all included studies.

Another important consideration is that the timing of LAAO relative to the most recent GI bleeding episode may be clinically relevant. Patients referred soon after a major hemorrhagic event may differ substantially from those undergoing LAAO after a prolonged period of stabilization and endoscopic treatment. Unfortunately, the currently available literature does not consistently report the temporal relationship between bleeding episodes, GI work-up, lesion treatment and device implantation. This limits interpretation of recurrence patterns after LAAO and makes it difficult to determine whether adverse outcomes are driven primarily by patient frailty, inadequate bleeding source control or the antithrombotic regimen chosen after the procedure. Future studies should therefore report bleeding etiology, success and durability of endoscopic or other source-directed treatment, the number and timing of previous bleeding episodes and whether the underlying lesion is reversible or remains associated with persistent recurrence risk. These variables are likely to influence both candidacy for LAAO and tolerance of postprocedural antithrombotic therapy. Recent outcome reports in patients with previous GI bleeding further support the need for such granularity, as adverse bleeding outcomes after LAAO appear to be strongly influenced by baseline clinical vulnerability and prior hemorrhagic burden [8,10].

When placed in the broader context of LAAO research, the findings of this review are consistent with the general concept that the procedure is most valuable in patients with a clinically unfavorable balance between stroke prevention and bleeding risk [45,46]. However, unlike unselected LAAO populations, patients with previous GI bleeding require a more nuanced interpretation of procedural success. In these patients, a technically successful implantation with no periprocedural complication does not necessarily translate into optimal long-term benefit if recurrent bleeding occurs during the period of postprocedural antithrombotic therapy. Accordingly, the effectiveness of LAAO in this subgroup should be considered not only in terms of implantation success and stroke prevention, but also in relation to the ability to safely navigate the postprocedural period without major recurrent hemorrhage. This view is supported by comparative and observational reports suggesting that patients with previous GI bleeding may achieve stroke reduction after LAAO, yet continue to carry a meaningful residual risk of subsequent bleeding events [8,10].

The evidence synthesized in this review also points to an important gap in guideline-relevant knowledge. Although contemporary consensus documents and clinical practice guidelines increasingly recognize LAAO as an option for patients with contraindications to long-term anticoagulation, they provide limited specific guidance for individuals with previous GI bleeding, particularly regarding postimplant antithrombotic therapy. This remains true despite the availability of recent consensus and guideline documents, which support LAAO in selected high-risk populations but do not provide detailed, GI bleeding-specific recommendations for postprocedural antithrombotic management [8,10,23]. In practice, this leaves clinicians to extrapolate from broader LAAO trials or from general antithrombotic principles, neither of which fully captures the complexity of this population. The absence of dedicated prospective data means that individualized clinical judgment remains essential, but also exposes substantial variation in care. For this reason, one of the most useful outputs of the present review is the rationale for a practical treatment algorithm that incorporates bleeding history, treatability of the bleeding source, thromboembolic risk and tolerance of short-term antithrombotic therapy [5,15].

Several future research priorities emerge from these findings. First, prospective studies focused specifically on patients with previous GI bleeding are needed to better define clinical outcomes after LAAO and to distinguish between different bleeding phenotypes. Second, comparative evaluation of postprocedural antithrombotic regimens in this population is urgently needed, particularly with respect to single antiplatelet therapy, abbreviated dual antiplatelet therapy and reduced-dose anticoagulant strategies. Third, future studies should more systematically report the source, severity, timing, and endoscopic management of prior GI bleeding, as these variables are likely to be critical modifiers of outcome. Finally, the development of structured multidisciplinary pathways for patient selection and follow-up may improve consistency of care and help refine individualized treatment strategies in routine clinical practice. These priorities align with recent reviews stressing the need for better evidence on regimen selection, DRT prevention and individualized postimplant care pathways [11,12,13,14].

The methodological limitations of the included observational studies have important implications for interpretation of the findings. Selection bias may have influenced which patients were referred for LAAO, as patients considered sufficiently stable to undergo the procedure may differ systematically from those managed conservatively or excluded from intervention. Residual confounding is also likely because bleeding severity, frailty, renal function, comorbidity burden, adequacy of bleeding-source control and choice of postprocedural antithrombotic therapy were not uniformly measured or adjusted for across studies. Consequently, observed associations between LAAO, recurrent bleeding and thromboembolic outcomes cannot be interpreted as causal effects, and apparently favorable outcomes may partly reflect patient selection and unmeasured baseline differences. This review should be interpreted in light of several limitations of the available evidence. Most published studies are observational and retrospective; moreover, the apparent aggregate sample size was driven almost entirely by one large registry, whereas the other four studies comprised only 1161 GI bleeding patients. This introduces potential selection bias, confounding, and inconsistency in outcome reporting. Definitions of GI bleeding, major bleeding, recurrent bleeding and antithrombotic regimens varied substantially across studies, limiting direct comparability. In many reports, patients with prior GI bleeding were included as part of broader high-bleeding-risk cohorts rather than as the sole focus of investigation, which further complicates interpretation. Follow-up duration was often limited, and detailed information regarding bleeding source, lesion treatment and therapy adjustment during follow-up was frequently incomplete. As a result, the conclusions of this review should be regarded as hypothesis-generating and clinically informative rather than definitive. In addition, the certainty of evidence was not formally assessed using GRADE and should therefore be interpreted in the context of predominantly observational data and substantial heterogeneity across studies. The same methodological limitations are evident in the currently available literature indexed in PubMed, where most evidence on prior GI bleeding after LAAO remains observational and heterogeneously reported [8,10,11,12,13].

Despite these limitations, the currently available observational literature suggests that LAAO may represent a reasonable therapeutic option in selected patients with AF and prior GI bleeding, although its net clinical benefit cannot be established with certainty from the available evidence. At the same time, the postprocedural period remains a major area of vulnerability, and antithrombotic management appears to be the key determinant of whether the theoretical benefit of the procedure is fully realized in practice. The present findings therefore support a shift from a purely procedure-centered perspective toward a broader management framework in which device implantation, bleeding source evaluation and individualized postprocedural therapy are considered inseparable components of care [11,12,13,14,15].

## 5. Clinical Implications and Proposed Treatment Algorithm

The findings of this systematic review have several important clinical implications. Although percutaneous LAAO appears to be a feasible and potentially beneficial strategy in patients with AF and prior GI bleeding, optimal outcomes in this population depend on more than procedural success alone [7,8,10]. Careful patient selection, structured evaluation of the bleeding history and individualized postprocedural antithrombotic management are central to maximizing benefit while minimizing harm [5,11,13].

A key practical message emerging from the available evidence is that prior GI bleeding should not be regarded as a uniform indication for LAAO, but rather as a heterogeneous clinical condition requiring individualized assessment [6,8,10]. The mechanism, severity, timing and treatability of the bleeding event are all likely to influence the risk-benefit profile of the procedure. This heterogeneity includes clinically relevant distinctions such as active or recently unresolved bleeding, treated reversible lesions, recurrent obscure bleeding or angiodysplasia, portal hypertensive bleeding, chronic anemia and the potential need for concomitant antiplatelet therapy, all of which may influence both candidacy for LAAO and postprocedural management. Patients with a clearly identified and successfully treated bleeding source may tolerate short-term postprocedural antithrombotic therapy relatively well, whereas those with recurrent, diffuse or non-correctable bleeding lesions may remain at substantial hemorrhagic risk even after device implantation. Therefore, the decision to proceed with LAAO should ideally be based on a multidisciplinary evaluation integrating cardiologic and gastroenterologic expertise [5,11,13].

From a therapeutic perspective, the most challenging phase in these patients is the postprocedural period, during which antithrombotic therapy is usually required to reduce the risk of DRT while endothelialization occurs [11,12,13]. The currently available literature does not support a single uniform postimplant regimen for patients with prior GI bleeding. Instead, the evidence suggests that treatment should be tailored according to the individual balance between thromboembolic risk, recurrent bleeding risk, procedural characteristics, and tolerance of short-term therapy [5,9,11,13,14]. In clinical practice, this means that postprocedural treatment intensity should be adapted not only to conventional stroke and bleeding scores, but also to the characteristics of the previous GI bleeding episode and the adequacy of source control.

In this context, an expert-opinion-derived practical framework may help structure clinical decision-making, while acknowledging that the available evidence is limited, predominantly observational and of low certainty, and therefore does not support rigid phenotype-specific pathway recommendations. First, before LAAO, the indication for stroke prevention should be confirmed and the reason for avoiding long-term OAC clearly established. Second, the prior GI bleeding event should be characterized in detail, including the bleeding source, severity, recurrence pattern, need for transfusion or hospitalization, endoscopic findings, and whether definitive treatment has been achieved. Third, patients should be stratified according to the likelihood of tolerating temporary postprocedural antithrombotic therapy. Those with a treated and apparently stable bleeding source may be candidates for a more conventional short-term strategy, whereas patients with persistent or poorly controlled bleeding susceptibility may require a more conservative regimen. Fourth, close follow-up during the endothelialization period is essential, with early reassessment in the event of anemia, recurrent GI symptoms, or evidence of device-related thrombosis [11,12,13].

**Figure 2:**
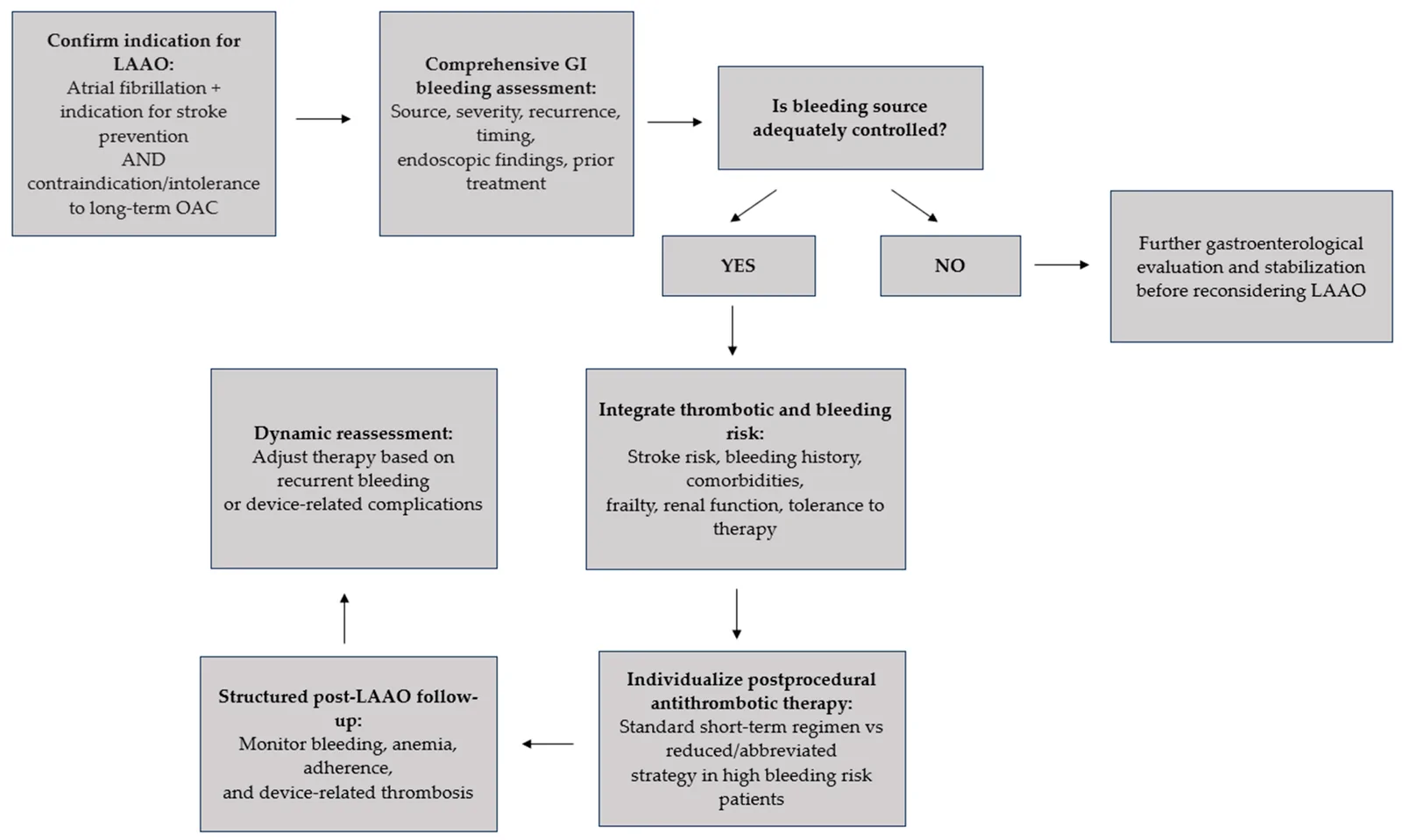
This figure presents an expert-informed and non-validated clinical framework. The general principles are informed by the systematic evidence synthesis, whereas specific management pathways and antithrombotic choices reflect expert interpretation supported by broader contextual literature. Abbreviations: GI, gastrointestinal; LAAO, left atrial appendage occlusion; OAC, oral anticoagulation.

Based on the limited GI bleeding-specific evidence and broader contextual literature, an expert-informed clinical framework may be proposed to structure individualized decision-making, as illustrated in Figure 2. This framework should be interpreted as a pragmatic clinical aid derived from low-certainty evidence rather than as a validated or evidence-based algorithm and it is not intended to translate all bleeding phenotypes into prescriptive management pathways. In patients with prior GI bleeding but a relatively controlled bleeding substrate and acceptable short-term bleeding risk, short-duration DAPT or another standard post-LAAO regimen may be considered, followed by de-escalation to SAPT when appropriate. In contrast, in patients with a very high risk of recurrent GI bleeding, SAPT or another minimized antithrombotic strategy may be more appropriate, provided that the risk of DRT is carefully monitored [11,12,13,14]. Patients with active, unresolved, or recently recurrent bleeding may require deferral of LAAO until the bleeding source is evaluated and stabilized, rather than immediate implantation under unfavorable conditions.

Importantly, the proposed framework should not be interpreted as a validated or evidence-based algorithm, nor as a substitute for clinical judgment, but rather as a structured and expert-opinion-derived approach reflecting the major considerations identified in the current literature and the limitations of a low-certainty evidence base. It is intended to support individualized reasoning across heterogeneous bleeding phenotypes rather than to define rigid management pathways.

The current evidence base remains insufficient to support rigid protocolization, and individualized care remains essential [5,11,13]. Nevertheless, an algorithmic approach may reduce unwarranted variability in practice and help clinicians move from a purely procedure-based view of LAAO toward a broader management model that incorporates bleeding-source control, postprocedural therapy planning, and multidisciplinary follow-up [5,11,13]. The proposed framework combines findings derived from the GI bleeding-specific evidence synthesis with expert interpretation informed by broader contextual LAAO literature and current clinical practice. The general principles of careful patient selection, detailed characterization of the bleeding source and individualized postprocedural antithrombotic management are supported by the available evidence. However, the specific sequencing of individual steps, the proposed stratification according to bleeding phenotype and the selection of particular antithrombotic regimens have not been prospectively validated in patients with prior GI bleeding. These elements should therefore be regarded as expert-opinion-derived and non-prescriptive.

### Expert-Informed Clinical Framework for Patients with Prior GI Bleeding Considered for LAAO

The proposed framework integrates findings from the GI bleeding-specific evidence synthesis with expert interpretation informed by broader contextual literature. Individual management steps have not been prospectively validated in patients with prior gastrointestinal bleeding and should therefore be considered non-prescriptive. For transparency, the elements of the framework are classified as either evidence-informed principles, when supported by findings consistently identified in the GI bleeding-specific evidence synthesis, or expert-opinion-derived recommendations, when based primarily on indirect evidence, broader contextual literature and multidisciplinary clinical reasoning. Formal high, moderate or low evidence grades were not assigned because a GRADE assessment was not performed and the available direct evidence was observational and heterogeneous.

**Step 1. Confirm indication for LAAO**

Patients should have atrial fibrillation with an indication for stroke prevention and a clinical scenario in which long-term oral anticoagulation is unsuitable, poorly tolerated or associated with unacceptable bleeding risk.

**Step 2. Characterize prior GI bleeding in detail**

The bleeding history should include bleeding source, severity, timing, recurrence, transfusion requirement, endoscopic findings and whether the lesion has been treated or remains at risk for rebleeding.

**Step 3. Assess bleeding-source control and reversibility**

Patients with a definitively treated and stabilized source of GI bleeding may be better candidates for LAAO followed by temporary antithrombotic therapy. In contrast, patients with active, obscure or recurrent bleeding should undergo further gastroenterological evaluation before implantation whenever feasible.

**Step 4. Evaluate competing thrombotic and bleeding risks**

Decision-making should integrate thromboembolic risk, prior bleeding burden, comorbidity profile, frailty, renal function and the expected ability to tolerate short-term antithrombotic therapy after implantation.

**Step 5. Individualize postprocedural antithrombotic therapy**

Patients at lower rebleeding risk may receive a more conventional short-term postimplant regimen, whereas those at very high bleeding risk may require an abbreviated or less intensive strategy. The choice should be individualized and ideally supported by multidisciplinary consensus.

**Step 6. Ensure close follow-up after implantation**

Early follow-up should focus on recurrent GI symptoms, anemia, occult bleeding, adherence to therapy and surveillance for device-related thrombosis or other complications. Therapy de-escalation should be considered as soon as clinically appropriate.

**Step 7. Reassess dynamically**

Management should remain flexible. Recurrent bleeding, new endoscopic findings or device-related complications may require modification of antithrombotic therapy and renewed multidisciplinary review.

From a broader clinical standpoint, this proposed framework emphasizes that the success of LAAO in patients with prior GI bleeding is determined not only in the catheterization laboratory, but also in the careful preparation before implantation and in the individualized management that follows. In this high-risk population, procedural success, bleeding prevention and thromboembolic protection should be viewed as interdependent treatment goals rather than separate outcomes [5,10,11,14]. This framework is intended to support clinical reasoning in a field where direct comparative evidence remains limited and should therefore be interpreted as expert-informed rather than formally validated. Accordingly, the framework is intended to provide a clinically structured but non-prescriptive approach, acknowledging that current evidence is insufficient to support phenotype-specific treatment pathways with high certainty.

## 6. Conclusions

Percutaneous left atrial appendage occlusion appears to be a feasible and potentially beneficial stroke prevention strategy in selected patients with atrial fibrillation and a history of gastrointestinal bleeding, particularly when long-term oral anticoagulation is contraindicated, poorly tolerated or associated with an unacceptable risk of recurrent hemorrhage. The available evidence suggests that LAAO may provide an acceptable balance between thromboembolic protection and bleeding avoidance in this high-risk population, although the current literature remains limited and is based predominantly on observational data [7,8,10].

A central finding of this review is that the clinical benefit of LAAO in patients with prior GI bleeding depends not only on procedural success, but also on the ability to safely navigate the postprocedural period. In this regard, postprocedural antithrombotic therapy remains one of the most important unresolved challenges. Current strategies are highly heterogeneous, reflecting the lack of dedicated evidence and the need for individualized treatment decisions based on bleeding history, thromboembolic risk and the treatability of the underlying GI lesion [5,9,11,13,14].

These findings support a patient-centered and multidisciplinary approach to LAAO in patients with previous GI bleeding. Careful gastroenterological evaluation, appropriate bleeding-source control, individualized selection of postprocedural antithrombotic therapy and close follow-up are likely to be essential for optimizing outcomes. Rather than viewing prior GI bleeding as a single uniform clinical category, future practice should recognize the heterogeneity of this population and tailor management accordingly [5,10,11,13].

Prospective studies specifically focused on patients with previous GI bleeding are needed to better define procedural outcomes, identify predictors of recurrent hemorrhage and determine the safest and most effective postimplant antithrombotic regimens. Until such evidence becomes available, individualized decision-making supported by multidisciplinary collaboration remains the most appropriate strategy for integrating LAAO into the management of this challenging patient population, while avoiding overgeneralization of benefit in clinically vulnerable subgroups [5,10,13]. Overall, these conclusions should be interpreted cautiously, as they are based predominantly on observational and heterogeneous evidence, with direct GI bleeding-specific data available only for a subset of the included studies, while the available randomized data remain indirect with respect to patients with prior GI bleeding. Given the small number of eligible studies, their observational design and substantial clinical and methodological heterogeneity, these findings should be considered low-certainty and hypothesis-generating.

## Figures and Tables

**Table 1:** Characteristics of included studies.

Study	Year	Country	Study Design	Sample Size	LAAO Device	Population	GI Bleeding	Evidence Category	Follow-Up	Main Outcome Reported
Lempereur et al. [14]	2017	International	Registry	1047 (151 prior major GI bleeding)	ACP	AF	Prior major	GI-specific	1.3 y	Stroke/bleeding
Kikuchi et al. [16]	2024	Japan	Retrospective	45 (19 prior GI bleeding)	WATCHMAN/FLX	AF	GI subgroup	GI subgroup	19 mo	Recurrent GI bleeding
Gallo et al. LOGIC [15]	2023	International	Registry	628 (358 prior GI bleeding)	Mixed	AF	Prior GI	GI subgroup	12 mo	Stroke/bleeding
Darden et al. [17]	2026	USA	Registry	250,779 (84,472 prior GI bleeding	WATCHMAN	AF	Prior GI	GI subgroup	1 y	GI bleeding
Preisendörfer et al. [27]	2026	USA	Matched cohort	1266 (633 LAAO; 633 OAC)	WATCHMAN/Amplatzer	AF	Prior major	GI-specific	30.9 mo	Recurrent GI bleeding

Abbreviations: ACP, Amplatzer Cardiac Plug; AF, atrial fibrillation; GI, gastrointestinal; LAAO, left atrial appendage occlusion; mo, months; OAC, oral anticoagulation; y, years.

**Table 2:** Study-level clinical outcomes and postprocedural antithrombotic regimens across the included studies.

Study	Evidence Category	GI-Specific Denominator	Follow-Up	Recurrent GI Bleeding	Stroke/Systemic Embolism	Device-Related Thrombosis	Mortality	Postprocedural Antithrombotic Regimen
Lempereur et al. [14]	a—Gastrointestinal bleeding-specific evidence	151 patients with prior major GI bleeding	Mean 1.3 years	Not reported separately; annual major bleeding rate 4.6%	Stroke/TIA annual rate 2.1%	NR	NR	DAPT/SAPT
Kikuchi et al. [16]	b—Gastrointestinal bleeding subgroup evidence	19 patients with prior GI bleeding	Median 19 months	7 post-LAAO GI bleeding events	NR	NR	NR	Variable; anticoagulation plus SAPT initially, followed by DAPT or aspirin-based therapy
Gallo et al. LOGIC [15]	b—Gastrointestinal bleeding subgroup evidence	358/628 patients with prior GI bleeding	12 months	NR	Stroke/TIA and systemic embolism reported; no significant increase with light antithrombotic regimen	NR	All-cause mortality reported; no significant difference between regimen groups	Variable; SAPT/no therapy vs. DAPT/anticoagulation
Darden et al. [17]	b—Gastrointestinal bleeding subgroup evidence	84,472/250,779 patients with prior GI bleeding	45 days and 1 year	Increased risk of GI bleeding at 45 days and 1 year	No significant difference in stroke risk	NR	Increased 1-year mortality in prior GI bleeding patients	Conservative regimens more common (no therapy, warfarin, DAPT)
Preisendörfer et al. [27]	a—Gastrointestinal bleeding-specific evidence	633 LAAO patients with previous major GI bleeding	Median 30.9 months	97.2/1000 person-years	Ischemic stroke/TIA 15.6/1000 person-years	NR	88.9/1000 person-years	Guideline-based postprocedural antithrombotic therapy

Note: Exact study-level outcome data are reported where available from the original publications. Because the definitions of recurrent gastrointestinal bleeding, thromboembolic events, device-related thrombosis, and mortality varied across studies, outcomes are presented according to the original study reports and were not formally harmonized. NR, not reported; DAPT, dual antiplatelet therapy; SAPT, single antiplatelet therapy; GI, gastrointestinal.

**Table 3:** Methodological appraisal of included studies.

Study	JBI Appraisal Summary	Main Methodological Considerations
Lempereur et al. [14]	Generally favorable	Residual confounding; incomplete reporting of follow-up
Kikuchi et al. [16]	Generally favorable	Small retrospective cohort; limited confounding adjustment
Gallo et al. LOGIC [15]	Generally favorable	Potential confounding by indication; follow-up reporting limitations
Darden et al. [17]	Favorable	Residual confounding inherent to registry design
Preisendörfer et al. [27]	Favorable	Potential residual/unmeasured confounding despite propensity matching

Abbreviations: Methodological appraisal was performed using the Joanna Briggs Institute Critical Appraisal Checklist for Cohort Studies. Overall appraisals were based on qualitative consideration of the individual checklist items and were not derived from numerical scoring thresholds. Detailed item-level judgments and supporting explanations are provided in Supplementary Table S4.

## Data Availability

No new data were created or analyzed in this study. Data sharing is not applicable to this article.

## Supplementary Materials

The following supporting information can be downloaded at https://www.mdpi.com/article/10.3390/biomedicines14081801/s1. Table S1. Full database-specific search strategies and numbers of records retrieved from each database; Table S2. Full-text articles excluded after eligibility assessment and reason for exclusion; Table S3. Detailed study-level gastrointestinal bleeding characteristics and postprocedural management variables; Table S4. JBI critical appraisal of included cohort studies; Table S5. Principal sources of clinical and methodological heterogeneity precluding quantitative synthesis.

## Abbreviations

The following abbreviations are used in this manuscript:

Abbreviation	Definition
AF	Atrial fibrillation
GI	Gastrointestinal
LAAO	Left atrial appendage occlusion
OAC	Oral anticoagulation
DOAC	Direct oral anticoagulant
DAPT	Dual antiplatelet therapy
SAPT	Single antiplatelet therapy
DRT	Device-related thrombosis
PRISMA	Preferred Reporting Items for Systematic Reviews and Meta-Analyses
ROBINS-I	Risk of Bias in Non-randomized Studies of Interventions
RoB 2	Cochrane Risk of Bias 2 tool

## References

[1] Hindricks G., Potpara T., Dagres N., Arbelo E., Bax J.J., Blomström-Lundqvist C., Boriani G., Castella M., Dan G.-A., Dilaveris P.E. (2021). Corrigendum to: 2020 ESC Guidelines for the Diagnosis and Management of Atrial Fibrillation Developed in Collaboration with the European Association for Cardio-Thoracic Surgery (EACTS): The Task Force for the Diagnosis and Management of Atrial Fibrillation of the European Society of Cardiology (ESC) Developed with the Special Contribution of the European Heart Rhythm Association (EHRA) of the ESC. Eur. Heart J..

[2] Joglar J.A., Chung M.K., Armbruster A.L., Benjamin E.J., Chyou J.Y., Cronin E.M., Deswal A., Eckhardt L.L., Goldberger Z.D., Gopinathannair R. (2024). 2023 ACC/AHA/ACCP/HRS Guideline for the Diagnosis and Management of Atrial Fibrillation: A Report of the American College of Cardiology/American Heart Association Joint Committee on Clinical Practice Guidelines. Circulation.

[3] Abraham N.S., Noseworthy P.A., Yao X., Sangaralingham L.R., Shah N.D. (2017). Gastrointestinal Safety of Direct Oral Anticoagulants: A Large Population-Based Study. Gastroenterology.

[4] Witt D.M., Delate T., Garcia D.A., Clark N.P., Hylek E.M., Ageno W., Dentali F., Crowther M.A. (2012). Risk of Thromboembolism, Recurrent Hemorrhage, and Death after Warfarin Therapy Interruption for Gastrointestinal Tract Bleeding. Arch. Intern. Med..

[5] Qureshi W., Mittal C., Patsias I., Garikapati K., Kuchipudi A., Cheema G., Elbatta M., Alirhayim Z., Khalid F. (2014). Restarting Anticoagulation and Outcomes after Major Gastrointestinal Bleeding in Atrial Fibrillation. Am. J. Cardiol..

[6] Stoddard M.F., Dawkins P.R., Prince C.R., Ammash N.M. (1995). Left Atrial Appendage Thrombus Is Not Uncommon in Patients with Acute Atrial Fibrillation and a Recent Embolic Event: A Transesophageal Echocardiographic Study. J. Am. Coll. Cardiol..

[7] Patti G., Pengo V., Marcucci R., Cirillo P., Renda G., Santilli F., Calabrò P., De Caterina A.R., Cavallari I., Ricottini E. (2017). The Left Atrial Appendage: From Embryology to Prevention of Thromboembolism. Eur. Heart J..

[8] Potpara T., Grygier M., Häusler K.G., Nielsen-Kudsk J.E., Berti S., Genovesi S., Marijon E., Boveda S., Tzikas A., Boriani G. (2024). Practical Guide on Left Atrial Appendage Closure for the Non-Implanting Physician: An International Consensus Paper. Europace.

[9] Landmesser U., Skurk C., Tzikas A., Falk V., Reddy V.Y., Windecker S. (2024). Left Atrial Appendage Closure for Stroke Prevention in Atrial Fibrillation: Current Status and Perspectives. Eur. Heart J..

[10] Goldsweig A.M., Glikson M., Joza J., Kavinsky C.J., Khalique O., Lakkireddy D., Mackensen G.B., Naccarelli G.V., Nair D.G., Saw J. (2025). 2025 SCAI/HRS Clinical Practice Guidelines on Transcatheter Left Atrial Appendage Occlusion. Heart Rhythm.

[11] Holmes D.R., Reddy V.Y., Turi Z.G., Doshi S.K., Sievert H., Buchbinder M., Mullin C.M., Sick P., PROTECT AF Investigators (2009). Percutaneous Closure of the Left Atrial Appendage versus Warfarin Therapy for Prevention of Stroke in Patients with Atrial Fibrillation: A Randomised Non-Inferiority Trial. Lancet.

[12] Reddy V.Y., Doshi S.K., Sievert H., Buchbinder M., Neuzil P., Huber K., Halperin J.L., Holmes D., PROTECT AF Investigators (2013). Percutaneous Left Atrial Appendage Closure for Stroke Prophylaxis in Patients with Atrial Fibrillation: 2.3-Year Follow-up of the PROTECT AF (Watchman Left Atrial Appendage System for Embolic Protection in Patients with Atrial Fibrillation) Trial. Circulation.

[13] Osmancik P., Herman D., Neuzil P., Hala P., Taborsky M., Kala P., Poloczek M., Stasek J., Haman L., Branny M. (2020). Left Atrial Appendage Closure Versus Direct Oral Anticoagulants in High-Risk Patients with Atrial Fibrillation. J. Am. Coll. Cardiol..

[14] Lempereur M., Aminian A., Freixa X., Gafoor S., Shakir S., Omran H., Berti S., Santoro G., Kefer J., Landmesser U. (2017). Left Atrial Appendage Occlusion in Patients with Atrial Fibrillation and Previous Major Gastrointestinal Bleeding (from the Amplatzer Cardiac Plug Multicenter Registry). Am. J. Cardiol..

[15] Gallo F., Ronco F., D’Amico G., Della Rocca D.G., Mazzone P., Bordignon S., Casu G., Giannini F., Berti S., Horton R.P. (2023). Clinical Outcomes of Left Atrial Appendage Occlusion in Patients with Previous Intracranial or Gastrointestinal Bleeding: Insights from the LOGIC (Left Atrial Appendage Occlusion in Patients with Gastrointestinal or IntraCranial Bleeding) International Multicenter Registry. Catheter. Cardiovasc. Interv..

[16] Kikuchi T., Kono Y., Nakagawa K., Okada H., Miyamoto M., Takaya Y., Hirata S., Inoo S., Kuraoka S., Okanoue S. (2024). Clinical Significance of Gastrointestinal Bleeding History in Patients Who Undergo Left Atrial Appendage Closure. JGH Open.

[17] Darden D., Katapadi A., Huang C.Y., Zimmerman S., Lakkireddy D., Kabra R., Pothineni N.V.K., Gopinathannair R., Munir M.B., Freeman J.V. (2026). Outcomes of Left Atrial Appendage Occlusion in Patients with Prior Gastrointestinal Bleeds. JACC Clin. Electrophysiol..

[18] Becic T., Jukić I., Prižmić P.Š., Matulić I., Đogaš H., Radić M., Radić J., Vuković J., Fabijanić D. (2026). Echocardiographic Guidance for Percutaneous Left Atrial Appendage Occlusion: A Systematic Review of Outcomes in High-Risk Populations Including Chronic Liver Disease and Prior Gastrointestinal Bleeding. Diagnostics.

[19] Galea R., Räber L. (2023). Antithrombotic Therapy after Percutaneous Left Atrial Appendage Closure: Evidence, Challenges and Future Directions. Rev. Cardiovasc. Med..

[20] Mesnier J., Cepas-Guillén P., Freixa X., Flores-Umanzor E., Hoang Trinh K., O’Hara G., Rodés-Cabau J. (2023). Antithrombotic Management After Left Atrial Appendage Closure: Current Evidence and Future Perspectives. Circ. Cardiovasc. Interv..

[21] Kramer A., Patti G., Nielsen-Kudsk J.E., Berti S., Korsholm K. (2024). Left Atrial Appendage Occlusion and Post-Procedural Antithrombotic Management. J. Clin. Med..

[22] Guduguntla V., Patton K.K. (2024). Rethinking Anticoagulation for Left Atrial Appendage Closure. JAMA Cardiol..

[23] Cronin E.M., Filby S., Field M.E., Huded C., Indik J.H., Sharma A., Armah C., Firestone S., Fix A.M., Senerth E. (2025). SCAI/HRS Technical Review on Transcatheter Left Atrial Appendage Occlusion. Heart Rhythm..

[24] Freeman J.V., Higgins A.Y., Wang Y., Du C., Friedman D.J., Daimee U.A., Minges K.E., Pereira L., Goldsweig A.M., Price M.J. (2022). Antithrombotic Therapy After Left Atrial Appendage Occlusion in Patients with Atrial Fibrillation. J. Am. Coll. Cardiol..

[25] Dukkipati S.R., Kar S., Holmes D.R., Doshi S.K., Swarup V., Gibson D.N., Maini B., Gordon N.T., Main M.L., Reddy V.Y. (2018). Device-Related Thrombus After Left Atrial Appendage Closure: Incidence, Predictors, and Outcomes. Circulation.

[26] Fauchier L., Cinaud A., Brigadeau F., Lepillier A., Pierre B., Abbey S., Fatemi M., Franceschi F., Guedeney P., Jacon P. (2018). Device-Related Thrombosis After Percutaneous Left Atrial Appendage Occlusion for Atrial Fibrillation. J. Am. Coll. Cardiol..

[27] Preisendörfer S., Ayub M.T., Sheth A., Wann D., Howard A., Thoma F.W., Zhu J., Jabbour G.Y., Singh M., Patel C.P. (2026). Left Atrial Appendage Occlusion in Patients with Atrial Fibrillation and Major Gastrointestinal Bleeding: Outcomes from a Multi-Hospital Health System. Europace.

[28] Glikson M., Wolff R., Hindricks G., Mandrola J., Camm A.J., Lip G.Y.H., Fauchier L., Betts T.R., Lewalter T., Saw J. (2020). EHRA/EAPCI Expert Consensus Statement on Catheter-Based Left Atrial Appendage Occlusion—An Update. Europace.

[29] Meier B., Blaauw Y., Khattab A.A., Lewalter T., Sievert H., Tondo C., Glikson M. (2014). Document Reviewers EHRA/EAPCI Expert Consensus Statement on Catheter-Based Left Atrial Appendage Occlusion. Europace.

[30] Sengupta N., Feuerstein J.D., Patwardhan V.R., Tapper E.B., Ketwaroo G.A., Thaker A.M., Leffler D.A. (2015). The Risks of Thromboembolism vs. Recurrent Gastrointestinal Bleeding after Interruption of Systemic Anticoagulation in Hospitalized Inpatients with Gastrointestinal Bleeding: A Prospective Study. Am. J. Gastroenterol..

[31] Majeed A., Kim Y.-K., Roberts R.S., Holmström M., Schulman S. (2010). Optimal Timing of Resumption of Warfarin after Intracranial Hemorrhage. Stroke.

[32] Boersma L.V., Ince H., Kische S., Pokushalov E., Schmitz T., Schmidt B., Gori T., Meincke F., Protopopov A.V., Betts T. (2019). Evaluating Real-World Clinical Outcomes in Atrial Fibrillation Patients Receiving the WATCHMAN Left Atrial Appendage Closure Technology: Final 2-Year Outcome Data of the EWOLUTION Trial Focusing on History of Stroke and Hemorrhage. Circ. Arrhythmia Electrophysiol..

[33] Holmes D.R., Kar S., Price M.J., Whisenant B., Sievert H., Doshi S.K., Huber K., Reddy V.Y. (2014). Prospective Randomized Evaluation of the Watchman Left Atrial Appendage Closure Device in Patients with Atrial Fibrillation versus Long-Term Warfarin Therapy: The PREVAIL Trial. J. Am. Coll. Cardiol..

[34] Freeman J.V., Varosy P., Price M.J., Slotwiner D., Kusumoto F.M., Rammohan C., Kavinsky C.J., Turi Z.G., Akar J., Koutras C. (2020). The NCDR Left Atrial Appendage Occlusion Registry. J. Am. Coll. Cardiol..

[35] Antúnez-Muiños P., López-Tejero S., Cepas-Guillén P., Mon-Noboa M., Ruiz-Nodar J.M., Andrés-Lalaguna L., Rivero F., Córdoba-Soriano J.G., Amat-Santos I.J., Caneiro-Queija B. (2024). A Comparison of Simplified or Conventional Antithrombotic Regimens after Left Atrial Appendage Closure in Patients at High Bleeding Risk: The PLATEBRISK Study. EuroIntervention.

[36] Llagostera-Martín M., Cainzos M., Salvatella N., Cubero-Gallego H., Mas-Stachurska A., Sánchez-Carpintero A., Tizón-Marcos H., Calvo-Fernández A., Molina L., Vaquerizo B. (2024). Single Antiplatelet Therapy after Left Atrial Appendage Closure in Patients with AF: Safety and Effectiveness. Rev. Esp. Cardiol..

[37] Della Rocca D.G., Magnocavallo M., Di Biase L., Mohanty S., Trivedi C., Tarantino N., Gianni C., Lavalle C., Van Niekerk C.J., Romero J. (2021). Half-Dose Direct Oral Anticoagulation Versus Standard Antithrombotic Therapy After Left Atrial Appendage Occlusion. JACC Cardiovasc. Interv..

[38] Enomoto Y., Gadiyaram V.K., Gianni C., Horton R.P., Trivedi C., Mohanty S., Di Biase L., Al-Ahmad A., Burkhardt J.D., Narula A. (2017). Use of Non-Warfarin Oral Anticoagulants Instead of Warfarin during Left Atrial Appendage Closure with the Watchman Device. Heart Rhythm.

[39] Kar S., Doshi S.K., Sadhu A., Horton R., Osorio J., Ellis C., Stone J., Shah M., Dukkipati S.R., Adler S. (2021). Primary Outcome Evaluation of a Next-Generation Left Atrial Appendage Closure Device: Results From the PINNACLE FLX Trial. Circulation.

[40] Lakkireddy D., Thaler D., Ellis C.R., Swarup V., Sondergaard L., Carroll J., Gold M.R., Hermiller J., Diener H.-C., Schmidt B. (2021). Amplatzer Amulet Left Atrial Appendage Occluder Versus Watchman Device for Stroke Prophylaxis (Amulet IDE): A Randomized, Controlled Trial. Circulation.

[41] Freixa X., Cruz-González I., Cepas-Guillén P., Millán X., Antúnez-Muiños P., Flores-Umanzor E., Asmarats L., Regueiro A., López-Tejero S., Li C.-H.P. (2024). Low-Dose Direct Oral Anticoagulation vs Dual Antiplatelet Therapy After Left Atrial Appendage Occlusion: The ADALA Randomized Clinical Trial. JAMA Cardiol..

[42] Rodés-Cabau J., Nombela-Franco L., Cruz-Gonzalez I., Hibbert B., Freixa X., Masson J.-B., Ibrahim R., Estevez-Loureiro R., Millan X., Kass M. (2025). Short-Term Anticoagulation Versus Dual Antiplatelet Therapy for Preventing Device Thrombosis Following Left Atrial Appendage Closure: The ANDES Randomized Clinical Trial. Circulation.

[43] Samaras A., Karakasis P., Feidakis A., Giannakoulas G., Fragakis N., Nielsen-Kudsk J.-E., Freixa X., Nair D.G., Freeman J.V., Bergmann M. (2026). Antithrombotic strategies and DOAC dosing following left atrial appendage occlusion: A network meta-analysis. Eur. Heart J.-Cardiovasc. Pharmacother..

[44] Sedaghat A., Vij V., Al-Kassou B., Gloekler S., Galea R., Fürholz M., Meier B., Valgimigli M., O’Hara G., Arzamendi D. (2021). Device-Related Thrombus After Left Atrial Appendage Closure: Data on Thrombus Characteristics, Treatment Strategies, and Clinical Outcomes From the EUROC-DRT-Registry. Circ. Cardiovasc. Interv..

[45] Osmancik P., Herman D., Neuzil P., Hala P., Taborsky M., Kala P., Poloczek M., Stasek J., Haman L., Branny M. (2022). 4-Year Outcomes After Left Atrial Appendage Closure Versus Nonwarfarin Oral Anticoagulation for Atrial Fibrillation. J. Am. Coll. Cardiol..

[46] Saw J., Inohara T., Gilhofer T., Uchida N., Pearce C., Dehghani P., Kass M., Ibrahim R., Morillo C., Wardell S. (2023). The Canadian WATCHMAN Registry for Percutaneous Left Atrial Appendage Closure. CJC Open.

