# Supplementary material for: Clinical Outcomes and Postprocedural Antithrombotic Management After Left Atrial Appendage Occlusion in Patients with Gastrointestinal Bleeding: A Systematic Review and Expert-Informed Clinical Framework
Source: Biomedicines. 2026 Aug 11;14(8):1801. doi: 10.3390/biomedicines14081801 (PMC13509920; doi:10.3390/biomedicines14081801)
Supplement: Supplementary file 1 [file biomedicines-14-01801-s001.zip › biomedicines-4457239-supplementary.pdf]

Supplementary Table S1. Full database-specific search strategies and numbers of records retrieved from each database.

Database	Date of search	Records retrieved	Search strategy
PubMed	27 April 2026	91	("Atrial Fibrillation"[Mesh] OR "atrial fibrillation"[Title/Abstract] OR AF[Title/Abstract]) AND ("left atrial appendage occlusion"[Title/Abstract] OR "left atrial appendage closure"[Title/Abstract] OR LAAO[Title/Abstract] OR LAAC[Title/Abstract] OR Watchman[Title/Abstract] OR Amulet[Title/Abstract] OR LAmbre[Title/Abstract]) AND ("Gastrointestinal Hemorrhage"[Mesh] OR "gastrointestinal bleeding"[Title/Abstract] OR "GI bleeding"[Title/Abstract] OR "gastrointestinal hemorrhage"[Title/Abstract] OR "gastrointestinal haemorrhage"[Title/Abstract] OR "gastrointestinal bleed"[Title/Abstract] OR "GI bleed"[Title/Abstract] OR "bleeding history"[Title/Abstract] OR "prior bleeding"[Title/Abstract]) AND ("Anticoagulants"[Mesh] OR "Platelet Aggregation Inhibitors"[Mesh] OR antithrombotic*[Title/Abstract] OR anticoagula*[Title/Abstract] OR antiplatelet*[Title/Abstract] OR "postprocedural therapy"[Title/Abstract] OR "post-procedural therapy"[Title/Abstract] OR "device-related thrombosis"[Title/Abstract] OR DRT[Title/Abstract])
Scopus	27 April 2026	272	TITLE-ABS-KEY(("atrial fibrillation" OR AF) AND ("left atrial appendage occlusion" OR "left atrial appendage closure" OR LAAO OR LAAC OR Watchman OR Amulet OR LAmbre) AND ("gastrointestinal bleeding" OR "GI bleeding" OR "gastrointestinal hemorrhage" OR "gastrointestinal haemorrhage" OR "gastrointestinal bleed" OR "GI bleed" OR "bleeding history" OR "prior bleeding") AND (antithrombotic* OR anticoagula* OR antiplatelet* OR "postprocedural therapy" OR "post-procedural therapy" OR "device-related thrombosis" OR DRT))
Web of Science	27 April 2026	264	TS=(("atrial fibrillation" OR AF) AND ("left atrial appendage occlusion" OR "left atrial appendage closure" OR LAAO OR LAAC OR Watchman OR Amulet OR LAmbre) AND ("gastrointestinal bleeding" OR "GI bleeding" OR "gastrointestinal hemorrhage" OR "gastrointestinal haemorrhage" OR "gastrointestinal bleed" OR "GI bleed" OR "bleeding history" OR "prior bleeding") AND (antithrombotic* OR anticoagula* OR antiplatelet* OR "postprocedural therapy" OR "post-procedural therapy" OR "device-related thrombosis" OR DRT))
Cochrane CENTRAL	27 April 2026	16	#1 MeSH descriptor: [Atrial Fibrillation] explode all trees #2 ("atrial fibrillation" OR AF):ti,ab,kw #3 ("left atrial appendage occlusion" OR "left atrial appendage closure" OR LAAO OR LAAC OR Watchman OR Amulet OR LAmbre):ti,ab,kw #4 MeSH descriptor: [Gastrointestinal Hemorrhage] explode all trees #5 ("gastrointestinal bleeding" OR "GI bleeding" OR "gastrointestinal hemorrhage" OR "gastrointestinal haemorrhage" OR "gastrointestinal bleed" OR "GI bleed" OR "bleeding history" OR "prior bleeding"):ti,ab,kw #6 MeSH descriptor: [Anticoagulants] explode all trees #7 MeSH descriptor: [Platelet Aggregation Inhibitors] explode all trees #8 (antithrombotic* OR anticoagula* OR antiplatelet* OR "postprocedural therapy" OR "post-procedural therapy" OR "device-related thrombosis" OR DRT):ti,ab,kw #9 (#1 OR #2) AND #3 AND (#4 OR #5) AND (#6 OR #7 OR #8)

Abbreviations: AF, atrial fibrillation; DRT, device-related thrombosis; GI, gastrointestinal; LAAC, left atrial appendage closure; LAAO, left atrial appendage occlusion.

Note: No language restrictions were applied during the initial search.

Supplementary Table S2. Full-text articles excluded after eligibility assessment and reason for exclusion

No.	References	Year	Reasons for exclusion
1	Hindricks, G.; Potpara, T.; Dagres, N.; Arbelo, E.; Bax, J.J.; Blomström-Lundqvist, C.; Boriani, G.; Castella, M.; Dan, G.-A.; Dilaveris, P.E.; et al. Corrigendum to: 2020 ESC Guidelines for the diagnosis and management of atrial fibrillation developed in collaboration with the European Association for Cardio-Thoracic Surgery (EACTS): The Task Force for the diagnosis and management of atrial fibrillation of the European Society of Cardiology (ESC) Developed with the special contribution of the European Heart Rhythm Association (EHRA) of the ESC. Eur. Heart J. 2021, 42, 4194. https://doi.org/10.1093/eurheartj/ehab648	2021	No LAAO-specific outcomes
2	Joglar, J.A.; Chung, M.K.; Armbruster, A.L.; Benjamin, E.J.; Chyou, J.Y.; Cronin, E.M.; Deswal, A.; Eckhardt, L.L.; Goldberger, Z.D.; Gopinathannair, R.; et al. 2023 ACC/AHA/ACCP/HRS Guideline for the Diagnosis and Management of Atrial Fibrillation: A Report of the American College of Cardiology/American Heart Association Joint Committee on Clinical Practice Guidelines. Circulation 2024, 149, e1–e156. https://doi.org/10.1161/CIR.0000000000001193	2024	No LAAO-specific outcomes
3	Potpara, T.; Grygier, M.; Häusler, K.G.; Nielsen-Kudsk, J.E.; Berti, S.; Genovesi, S.; Marijon, E.; Boveda, S.; Tzikas, A.; Boriani, G.; et al. Practical guide on left atrial appendage closure for the non-implanting physician: An international consensus paper. Europace 2024, 26, euae035. https://doi.org/10.1093/europace/euae035	2024	No LAAO-specific outcomes
4	Landmesser, U.; Skurk, C.; Tzikas, A.; Falk, V.; Reddy, V.Y.; Windecker, S. Left atrial appendage closure for stroke prevention in atrial fibrillation: Current status and perspectives. Eur. Heart J. 2024, 45, 2914–2932. https://doi.org/10.1093/eurheartj/ehae398	2024	Reviews/editorials/case reports
5	Goldsweig, A.M.; Glikson, M.; Joza, J.; Kavinsky, C.J.; Khalique, O.; Lakkireddy, D.; Mackensen, G.B.; Naccarelli, G.V.; Nair, D.G.; Saw, J.; et al. 2025 SCAI/HRS clinical practice guidelines on transcatheter left atrial appendage occlusion. Heart Rhythm 2025, 22, e1048–e1063. https://doi.org/10.1016/j.hrthm.2025.05.048	2025	No LAAO-specific outcomes
6	Cronin, E.M.; Filby, S.; Field, M.E.; Huded, C.; Indik, J.H.; Sharma, A.; Armah, C.; Firestone, S.; Fix, A.M.; Senerth, E.; et al. SCAI/HRS technical review on transcatheter left atrial appendage occlusion. Heart Rhythm 2025, 22, e1064–e1074. https://doi.org/10.1016/j.hrthm.2025.05.049	2025	No LAAO-specific outcomes
7	Galea, R.; Räber, L. Antithrombotic therapy after percutaneous left atrial appendage closure: Evidence, challenges and future directions. Rev. Cardiovasc. Med. 2023, 24, 343. https://doi.org/10.31083/j.rcm2412343	2023	Reviews/editorials/case reports
8	Mesnier, J.; Cepas-Guillén, P.; Freixa, X.; Flores-Umanzor, E.; Trinh, K.H.; O'Hara, G.; Rodés-Cabau, J. Antithrombotic Management after Left Atrial Appendage Closure: Current Evidence and Future Perspectives. *Circ. Cardiovasc. Interv.* 2023, *16*, e012812. https://doi.org/10.1161/CIRCINTERVENTIONS.122.012812	2023	Reviews/editorials/case reports
9	Kramer, A.; Patti, G.; Nielsen-Kudsk, J.E.; Berti, S.; Korsholm, K. Left Atrial Appendage Occlusion and Post-procedural Antithrombotic Management. *J. Clin. Med.* 2024, *13*, 803. https://doi.org/10.3390/jcm13030803	2024	Reviews/editorials/case reports
10	Guduguntla, V.; Patton, K.K. Rethinking Anticoagulation for Left Atrial Appendage Closure. *JAMA Cardiol.* 2024, *9*, 927. https://doi.org/10.1001/jamacardio.2024.2390	2024	Reviews/editorials/case reports
11	Raffo, C.; Greco, A.; Capodanno, D. Antithrombotic Therapy after Left Atrial Appendage Occlusion. *Expert Rev. Cardiovasc. Ther.* 2025, *23*, 141–152. https://doi.org/10.1080/14779072.2025.2486154	2025	Reviews/editorials/case reports
12	Stoddard, M.F.; Dawkins, P.R.; Prince, C.R.; Ammash, N.M. Left Atrial Appendage Thrombus Is Not Uncommon in Patients with Acute Atrial Fibrillation and a Recent Embolic Event: A Transesophageal Echocardiographic Study. *J. Am. Coll. Cardiol.* 1995, *25*, 452–459. https://doi.org/10.1016/0735-1097(94)00396-8	1995	No LAAO-specific outcomes
13	Patti, G.; Pengo, V.; Marcucci, R.; Cirillo, P.; Renda, G.; Santilli, F.; Calabrò, P.; De Caterina, A.R.; Cavallari, I.; Ricottini, E.; et al. The Left Atrial Appendage: From Embryology to Prevention of Thromboembolism. *Eur. Heart J.* 2017, *38*, 877–887. https://doi.org/10.1093/eurheartj/ehw159	2017	Reviews/editorials/case reports
14	Abraham, N.S.; Noseworthy, P.A.; Yao, X.; Sangaralingham, L.R.; Shah, N.D. Gastrointestinal Safety of Direct Oral Anticoagulants: A Large Population-Based Study. *Gastroenterology* 2017, *152*, 1014–1022.e1. https://doi.org/10.1053/j.gastro.2016.12.018	2017	No LAAO-specific outcomes
15	Witt, D.M.; Delate, T.; Garcia, D.A.; Clark, N.P.; Hylek, E.M.; Ageno, W.; Dentali, F.; Crowther, M.A. Risk of Thromboembolism, Recurrent Hemorrhage, and Death after Warfarin Therapy Interruption for	2012	No LAAO-specific outcomes

No.	References	Year	Reasons for exclusion
	Gastrointestinal Tract Bleeding. *Arch. Intern. Med.* 2012, *172*, 1484–1491. https://doi.org/10.1001/archinternmed.2012.4261		
16	Qureshi, W.; Mittal, C.; Patsias, I.; Garikapati, K.; Kuchipudi, A.; Cheema, G.; Elbatta, M.; Alirhayim, Z.; Khalid, F. Restarting Anticoagulation and Outcomes after Major Gastrointestinal Bleeding in Atrial Fibrillation. *Am. J. Cardiol.* 2014, *113*, 662–668. https://doi.org/10.1016/j.amjcard.2013.10.044	2014	No LAAO-specific outcomes
17	Proietti, M.; Romiti, G.F.; Romanazzi, I.; Farcomeni, A.; Staerk, L.; Nielsen, P.B.; Lip, G.Y.H. Restarting Oral Anticoagulant Therapy after Major Bleeding in Atrial Fibrillation: A Systematic Review and Meta-Analysis. *Int. J. Cardiol.* 2018, *261*, 84–91. https://doi.org/10.1016/j.ijcard.2018.03.053	2018	No LAAO-specific outcomes
18	Holmes, D.R.; Reddy, V.Y.; Turi, Z.G.; Doshi, S.K.; Sievert, H.; Buchbinder, M.; Mullin, C.M.; Sick, P.; PROTECT AF Investigators. Percutaneous Closure of the Left Atrial Appendage versus Warfarin Therapy for Prevention of Stroke in Patients with Atrial Fibrillation: A Randomised Non-Inferiority Trial. *Lancet* 2009, *374*, 534–542. https://doi.org/10.1016/S0140-6736(09)61343-X	2009	No specific GI bleeding population
19	Reddy, V.Y.; Doshi, S.K.; Sievert, H.; Buchbinder, M.; Neuzil, P.; Huber, K.; Halperin, J.L.; Holmes, D.; PROTECT AF Investigators. Percutaneous Left Atrial Appendage Closure for Stroke Prophylaxis in Patients with Atrial Fibrillation: 2.3-Year Follow-up of the PROTECT AF (Watchman Left Atrial Appendage System for Embolic Protection in Patients with Atrial Fibrillation) Trial. *Circulation* 2013, *127*, 720–729. https://doi.org/10.1161/CIRCULATIONAHA.112.114389	2013	No specific GI bleeding population
20	Holmes, D.R.; Kar, S.; Price, M.J.; Whisenant, B.; Sievert, H.; Doshi, S.K.; Huber, K.; Reddy, V.Y. Prospective Randomized Evaluation of the Watchman Left Atrial Appendage Closure Device in Patients with Atrial Fibrillation versus Long-Term Warfarin Therapy: The PREVAIL Trial. *J. Am. Coll. Cardiol.* 2014, *64*, 1–12. https://doi.org/10.1016/j.jacc.2014.04.029	2014	No specific GI bleeding population
21	Reddy, V.Y.; Möbius-Winkler, S.; Miller, M.A.; Neuzil, P.; Schuler, G.; Wiebe, J.; Sick, P.; Sievert, H. Left Atrial Appendage Closure with the Watchman Device in Patients with a Contraindication for Oral Anticoagulation: The ASAP Study (ASA Plavix Feasibility Study with Watchman Left Atrial Appendage Closure Technology). *J. Am. Coll. Cardiol.* 2013, *61*, 2551–2556. https://doi.org/10.1016/j.jacc.2013.03.035	2013	No specific GI bleeding population
22	Reddy, V.Y.; Gibson, D.N.; Kar, S.; O'Neill, W.; Doshi, S.K.; Horton, R.P.; Buchbinder, M.; Gordon, N.T.; Holmes, D.R. Post-Approval U.S. Experience with Left Atrial Appendage Closure for Stroke Prevention in Atrial Fibrillation. *J. Am. Coll. Cardiol.* 2017, *69*, 253–261. https://doi.org/10.1016/j.jacc.2016.10.010	2017	No specific GI bleeding population
23	Freeman, J.V.; Varosy, P.; Price, M.J.; Slotwiner, D.; Kusumoto, F.M.; Rammohan, C.; Kavinsky, C.J.; Turi, Z.G.; Akar, J.; Koutras, C.; et al. The NCDR Left Atrial Appendage Occlusion Registry. *J. Am. Coll. Cardiol.* 2020, *75*, 1503–1518. https://doi.org/10.1016/j.jacc.2019.12.040	2020	Insufficient clinically relevant GI bleeding-specific outcome data
24	Price, M.J.; Slotwiner, D.; Du, C.; Freeman, J.V.; Turi, Z.; Rammohan, C.; Kusumoto, F.M.; Kavinsky, C.; Akar, J.; Koutras, C.; et al. Clinical Outcomes at 1 Year following Transcatheter Left Atrial Appendage Occlusion in the United States. *JACC Cardiovasc. Interv.* 2022, *15*, 741–750. https://doi.org/10.1016/j.jcin.2022.02.009	2022	No specific GI bleeding population
25	Freeman, J.V.; Higgins, A.Y.; Wang, Y.; Du, C.; Friedman, D.J.; Daimee, U.A.; Minges, K.E.; Pereira, L.; Goldsweig, A.M.; Price, M.J.; et al. Antithrombotic Therapy after Left Atrial Appendage Occlusion in Patients with Atrial Fibrillation. *J. Am. Coll. Cardiol.* 2022, *79*, 1785–1798. https://doi.org/10.1016/j.jacc.2022.02.047	2022	No specific GI bleeding population
26	Reinhardt, S.W.; Gibson, D.N.; Hsu, J.C.; Kapadia, S.R.; Yeh, R.W.; Price, M.J.; Piccini, J.P.; Nair, D.G.; Christen, T.; Allocco, D.J.; et al. Anticoagulation Alone vs Anticoagulation plus Aspirin or DAPT following Left Atrial Appendage Occlusion. *J. Am. Coll. Cardiol.* 2024, *84*, 889–900. https://doi.org/10.1016/j.jacc.2024.05.067	2024	No specific GI bleeding population
27	Dukkipati, S.R.; Kar, S.; Holmes, D.R.; Doshi, S.K.; Swarup, V.; Gibson, D.N.; Maini, B.; Gordon, N.T.; Main, M.L.; Reddy, V.Y. Device-Related Thrombus after Left Atrial Appendage Closure: Incidence, Predictors, and Outcomes. *Circulation* 2018, *138*, 874–885. https://doi.org/10.1161/CIRCULATIONAHA.118.035090	2018	Insufficient clinically relevant GI bleeding-specific outcome data
28	Fauchier, L.; Cinaud, A.; Brigadeau, F.; Lepillier, A.; Pierre, B.; Abbey, S.; Fatemi, M.; Franceschi, F.; Guedeney, P.; Jacon, P.; et al. Device-Related Thrombosis after Percutaneous Left Atrial Appendage	2018	Insufficient clinically relevant GI bleeding-specific outcome data

No.	References	Year	Reasons for exclusion
	Occlusion for Atrial Fibrillation. *J. Am. Coll. Cardiol.* 2018, *71*, 1528–1536. https://doi.org/10.1016/j.jacc.2018.01.076		
29	Saw, J.; Tzikas, A.; Shakir, S.; Gafoor, S.; Omran, H.; Nielsen-Kudsk, J.E.; Kefer, J.; Aminian, A.; Berti, S.; Santoro, G.; et al. Incidence and Clinical Impact of Device-Associated Thrombus and Peri-Device Leak following Left Atrial Appendage Closure with the Amplatzer Cardiac Plug. *JACC Cardiovasc. Interv.* 2017, *10*, 391–399. https://doi.org/10.1016/j.jcin.2016.11.029	2017	Insufficient clinically relevant GI bleeding-specific outcome data
30	Korsholm, K.; Nielsen, K.M.; Jensen, J.M.; Jensen, H.K.; Andersen, G.; Nielsen-Kudsk, J.E. Transcatheter Left Atrial Appendage Occlusion in Patients with Atrial Fibrillation and a High Bleeding Risk Using Aspirin Alone for Post-Implant Antithrombotic Therapy. *EuroIntervention* 2017, *12*, 2075–2082. https://doi.org/10.4244/EIJ-D-16-00726	2017	No specific GI bleeding population
31	Nielsen-Kudsk, J.E.; Korsholm, K.; Damgaard, D.; Valentin, J.B.; Diener, H.-C.; Camm, A.J.; Johnsen, S.P. Clinical Outcomes Associated with Left Atrial Appendage Occlusion versus Direct Oral Anticoagulation in Atrial Fibrillation. *JACC Cardiovasc. Interv.* 2021, *14*, 69–78. https://doi.org/10.1016/j.jcin.2020.09.051	2021	No specific GI bleeding population
32	Tzikas, A.; Shakir, S.; Gafoor, S.; Omran, H.; Berti, S.; Santoro, G.; Kefer, J.; Landmesser, U.; Nielsen-Kudsk, J.E.; Cruz-Gonzalez, I.; et al. Left Atrial Appendage Occlusion for Stroke Prevention in Atrial Fibrillation: Multicentre Experience with the AMPLATZER Cardiac Plug. *EuroIntervention* 2016, *11*, 1170–1179. https://doi.org/10.4244/EIJY15M01_06	2016	No specific GI bleeding population
33	Urena, M.; Rodés-Cabau, J.; Freixa, X.; Saw, J.; Webb, J.G.; Freeman, M.; Horlick, E.; Osten, M.; Chan, A.; Marquis, J.-F.; et al. Percutaneous Left Atrial Appendage Closure with the AMPLATZER Cardiac Plug Device in Patients with Nonvalvular Atrial Fibrillation and Contraindications to Anticoagulation Therapy. *J. Am. Coll. Cardiol.* 2013, *62*, 96–102. https://doi.org/10.1016/j.jacc.2013.02.089	2013	No specific GI bleeding population
34	López-Mínguez, J.R.; Eldoayen-Gragera, J.; González-Fernández, R.; Fernández-Vegas, C.; Fuentes-Cañamero, M.E.; Millán-Nuñez, V.; Nogales-Asensio, J.M.; Martínez-Naharro, A.; Sánchez-Giralt, S.; Doblado-Calatrava, M.; et al. Immediate and One-Year Results in 35 Consecutive Patients after Closure of Left Atrial Appendage with the Amplatzer Cardiac Plug. *Rev. Esp. Cardiol. Engl. Ed.* 2013, *66*, 90–97. https://doi.org/10.1016/j.rec.2012.04.017	2013	Insufficient clinically relevant GI bleeding-specific outcome data
35	Cruz-González, I.; González-Ferreiro, R.; Freixa, X.; Gafoor, S.; Shakir, S.; Omran, H.; Berti, S.; Santoro, G.; Kefer, J.; Landmesser, U.; et al. Left Atrial Appendage Occlusion for Stroke despite Oral Anticoagulation (Resistant Stroke). Results from the Amplatzer Cardiac Plug Registry. *Rev. Esp. Cardiol. Engl. Ed.* 2020, *73*, 28–34. https://doi.org/10.1016/j.rec.2019.02.013	2020	No specific GI bleeding population
36	Berti, S.; Santoro, G.; Brscic, E.; Montorfano, M.; Vignali, L.; Danna, P.; Tondo, C.; D'Amico, G.; Stabile, A.; Saccà, S.; et al. Left Atrial Appendage Closure Using AMPLATZER™ Devices: A Large, Multicenter, Italian Registry. *Int. J. Cardiol.* 2017, *248*, 103–107. https://doi.org/10.1016/j.ijcard.2017.07.052	2017	No specific GI bleeding population
37	Kleinecke, C.; Park, J.-W.; Gödde, M.; Zintl, K.; Schnupp, S.; Brachmann, J. Twelve-Month Follow-Up of Left Atrial Appendage Occlusion with Amplatzer Amulet. *Cardiol. J.* 2017, *24*, 131–138. https://doi.org/10.5603/CJ.a2017.0017	2017	No specific GI bleeding population
38	Park, J.-W.; Bethencourt, A.; Sievert, H.; Santoro, G.; Meier, B.; Walsh, K.; Lopez-Minguez, J.R.; Meerkin, D.; Valdés, M.; Ormerod, O.; et al. Left Atrial Appendage Closure with Amplatzer Cardiac Plug in Atrial Fibrillation: Initial European Experience. *Catheter Cardiovasc. Interv.* 2011, *77*, 700–706. https://doi.org/10.1002/ccd.22764	2011	Insufficient clinically relevant GI bleeding-specific outcome data
39	Chun, K.R.J.; Bordignon, S.; Urban, V.; Perrotta, L.; Dugo, D.; Fürnkranz, A.; Nowak, B.; Schmidt, B. Left Atrial Appendage Closure Followed by 6 Weeks of Antithrombotic Therapy: A Prospective Single-Center Experience. *Heart Rhythm* 2013, *10*, 1792–1799. https://doi.org/10.1016/j.hrthm.2013.08.025	2013	No specific GI bleeding population
40	Søndergaard, L.; Wong, Y.-H.; Reddy, V.Y.; Boersma, L.V.A.; Bergmann, M.W.; Doshi, S.; Kar, S.; Sievert, H.; Wehrenberg, S.; Stein, K.; et al. Propensity-Matched Comparison of Oral Anticoagulation versus Antiplatelet Therapy after Left Atrial Appendage Closure with WATCHMAN. *JACC Cardiovasc. Interv.* 2019, *12*, 1055–1063. https://doi.org/10.1016/j.jcin.2019.04.004	2019	No specific GI bleeding population
41	Maksym, J.; Scisło, P.; Kapłon-Cieślicka, A.; Huczek, Z.; Marchel, M.; Kochman, J.; Zbroński, K.; Opolski, G.; Grabowski, M.; Mazurek, T. Incidence and Predictors of Device-Related Thrombus after Left Atrial Appendage Closure with Watchman Device. *Postepy Kardiol. Interwencyjnej* 2024, *20*, 164–171. https://doi.org/10.5114/aic.2024.140315	2024	Insufficient clinically relevant GI bleeding-specific outcome data

No.	References	Year	Reasons for exclusion
42	Reddy, V.Y.; Doshi, S.K.; Kar, S.; Gibson, D.N.; Price, M.J.; Huber, K.; Horton, R.P.; Buchbinder, M.; Neuzil, P.; Gordon, N.T.; et al. 5-Year Outcomes After Left Atrial Appendage Closure: From the PREVAIL and PROTECT AF Trials. *J. Am. Coll. Cardiol.* 2017, *70*, 2964–2975. https://doi.org/10.1016/j.jacc.2017.10.021.	2017	Insufficient data/duplicate cohorts
43	Brouwer, T.F.; Whang, W.; Kuroki, K.; Halperin, J.L.; Reddy, V.Y. Net Clinical Benefit of Left Atrial Appendage Closure Versus Warfarin in Patients With Atrial Fibrillation: A Pooled Analysis of the Randomized PROTECT-AF and PREVAIL Studies. *J. Am. Heart Assoc.* 2019, *8*, e013525. https://doi.org/10.1161/JAHA.119.013525	2019	Insufficient data/duplicate cohorts
44	Plicht, B.; Konorza, T.F.M.; Kahlert, P.; Al-Rashid, F.; Kaelsch, H.; Jánosi, R.A.; Buck, T.; Bachmann, H.S.; Siffert, W.; Heusch, G.; et al. Risk Factors for Thrombus Formation on the Amplatzer Cardiac Plug After Left Atrial Appendage Occlusion. *JACC Cardiovasc. Interv.* 2013, *6*, 606–613. https://doi.org/10.1016/j.jcin.2013.02.014	2013	Insufficient clinically relevant GI bleeding-specific outcome data
45	Rodés-Cabau, J.; Nombela-Franco, L.; Cruz-González, I.; Hibbert, B.; Freixa, X.; Masson, J.-B.; Ibrahim, R.; Estevez-Loureiro, R.; Millan, X.; Kass, M.; et al. Short-Term Anticoagulation versus Dual Antiplatelet Therapy for Preventing Device Thrombosis following Left Atrial Appendage Closure: The ANDES Randomized Clinical Trial. *Circulation* 2025, *152*, 1759–1768. https://doi.org/10.1161/CIRCULATIONAHA.125.077469	2025	No specific GI bleeding population
46	Carvalho, P.E.P.; Gewehr, D.M.; Miyawaki, I.A.; Nogueira, A.; Felix, N.; Garot, P.; Darmon, A.; Mazzone, P.; Preda, A.; Nascimento, B.R.; et al. Network Meta-Analysis of Initial Antithrombotic Regimens after Left Atrial Appendage Occlusion. *J. Am. Coll. Cardiol.* 2023, *82*, 1765–1773. https://doi.org/10.1016/j.jacc.2023.08.010	2023	Insufficient data/duplicate cohorts
47	Franciulli, M.; De Martino, G.; Librera, M.; Desoky, A.; Mariniello, A.; Iavazzo, A.; Scigliano, F.; Esposito, G.; Chiariello, L. Stand-Alone Thoracoscopic Left Atrial Appendage Closure in Nonvalvular Atrial Fibrillation Patients at High Bleeding Risk. *Innovations* 2020, *15*, 541–546. https://doi.org/10.1177/1556984520960116	2020	Insufficient data/duplicate cohorts
48	Rose, D.Z.; DiGiorgi, P.; Ramlawi, B.; Pulungan, Z.; Teigland, C.; Calkins, H. Minimally Invasive Epicardial Surgical Left Atrial Appendage Exclusion for Atrial Fibrillation Patients at High Risk for Stroke and for Bleeding. *Heart Rhythm* 2024, *21*, 771–779. https://doi.org/10.1016/j.hrthm.2024.01.046	2024	Insufficient data/duplicate cohorts
49	Mesnier, J.; Cepas-Guillén, P.; Freixa, X.; Flores-Umanzor, E.; Hoang Trinh, K.; O'Hara, G.; Rodés-Cabau, J. Antithrombotic Management After Left Atrial Appendage Closure: Current Evidence and Future Perspectives. *Circ Cardiovasc Interv* **2023**, *16*, e012812, doi:10.1161/CIRCINTERVENTIONS.122.012812.	2024	No separately extractable GI bleeding-specific outcomes
50	Antúnez-Muiños, P.; López-Tejero, S.; Cepas-Guillén, P.; Mon-Noboa, M.; Ruiz-Nodar, J.M.; Andrés-Lalaguna, L.; Rivero, F.; Córdoba-Soriano, J.G.; Amat-Santos, I.J.; Caneiro-Queija, B.; et al. A Comparison of Simplified or Conventional Antithrombotic Regimens after Left Atrial Appendage Closure in Patients at High Bleeding Risk: The PLATEBRISK Study. *EuroIntervention* **2024**, *20*, 1018–1028, doi:10.4244/EIJ-D-24-00116.	2024	No separately extractable GI bleeding-specific outcomes
51	Llagostera-Martín, M.; Cainzos, M.; Salvatella, N.; Cubero-Gallego, H.; Mas-Stachurska, A.; Sánchez-Carpintero, A.; Tizón-Marcos, H.; Calvo-Fernández, A.; Molina, L.; Vaquerizo, B. Single Antiplatelet Therapy after Left Atrial Appendage Closure in Patients with AF: Safety and Effectiveness. *Rev Esp Cardiol (Engl Ed)* **2024**, *77*, 150–157, doi:10.1016/j.rec.2023.06.020.	2024	No separately extractable GI bleeding-specific outcomes
52	Della Rocca, D.G.; Magnocavallo, M.; Di Biase, L.; Mohanty, S.; Trivedi, C.; Tarantino, N.; Gianni, C.; Lavalle, C.; Van Niekerk, C.J.; Romero, J.; et al. Half-Dose Direct Oral Anticoagulation Versus Standard Antithrombotic Therapy After Left Atrial Appendage Occlusion. *JACC Cardiovasc Interv* **2021**, *14*, 2353–2364, doi:10.1016/j.jcin.2021.07.031.	2021	No separately extractable GI bleeding-specific outcomes
53	Boersma, L.V.; Ince, H.; Kische, S.; Pokushalov, E.; Schmitz, T.; Schmidt, B.; Gori, T.; Meincke, F.; Protopopov, A.V.; Betts, T.; et al. Evaluating Real-World Clinical Outcomes in Atrial Fibrillation Patients Receiving the WATCHMAN Left Atrial Appendage Closure Technology: Final 2-Year Outcome Data of the EWOLUTION Trial Focusing on History of Stroke and Hemorrhage. *Circ Arrhythm Electrophysiol* **2019**, *12*, e006841, doi:10.1161/CIRCEP.118.006841.	2019	No separately extractable GI bleeding-specific outcomes

No.	References	Year	Reasons for exclusion
54	Osmancik, P.; Herman, D.; Neuzil, P.; Hala, P.; Taborsky, M.; Kala, P.; Poloczek, M.; Stasek, J.; Haman, L.; Branny, M.; et al. 4-Year Outcomes After Left Atrial Appendage Closure Versus Nonwarfarin Oral Anticoagulation for Atrial Fibrillation. *J Am Coll Cardiol* **2022**, *79*, 1–14, doi:10.1016/j.jacc.2021.10.023.	2022	No separately extractable GI bleeding-specific outcomes
55	Saw, J.; Inohara, T.; Gilhofer, T.; Uchida, N.; Pearce, C.; Dehghani, P.; Kass, M.; Ibrahim, R.; Morillo, C.; Wardell, S.; et al. The Canadian WATCHMAN Registry for Percutaneous Left Atrial Appendage Closure. *CJC Open* **2023**, *5*, 522–529, doi:10.1016/j.cjco.2023.03.016.	2023	No separately extractable GI bleeding-outcomes

Supplementary Table S3. Detailed study-level gastrointestinal bleeding characteristics and postprocedural management variables.

Study	GI bleeding source	Bleeding severity	Timing of prior GI bleeding	Endoscopic findings / treatment	Postprocedural antithrombotic regimen	Therapy modification during follow-up	Comment
Lempereur et al. [14]	Prior major gastrointestinal bleeding	Major GI bleeding	Not clearly reported	Not reported	DAPT or SAPT	Not reported	GI bleeding-specific cohort; limited GI source characterization
Kikuchi et al. [16]	Predominantly intestinal angiodysplasia and obscure GI bleeding	Recurrent clinically relevant GI bleeding	Prior bleeding before LAAO; exact interval not reported	Endoscopic evaluation performed in most patients	Initial anticoagulation plus SAPT, followed by DAPT or aspirin-based therapy	Antithrombotic regimen adjusted after recurrent bleeding events	One of the most detailed GI bleeding-specific analyses
Gallo et al. LOGIC [15]	Previous gastrointestinal bleeding broadly defined	Previous major bleeding history	Not reported	Not reported	Variable; SAPT/no therapy vs conventional therapy	Simplified regimens used in selected high-risk patients	GI bleeding analyzed as a major bleeding subgroup
Darden et al. [17]	Prior GI bleeding registry phenotype	Previous GI bleeding associated with increased bleeding risk	Evaluated before LAAO implantation	Not reported	Conservative regimens more commonly used in prior GI bleeding patients	Frequent use of no therapy, warfarin only, or DAPT	Large national registry analysis
Preisendörfer et al. [27]	Previous major gastrointestinal bleeding	Major GI bleeding requiring hospitalization	Prior bleeding before LAAO	Not consistently reported	Guideline-based postprocedural therapy	Not specifically reported	Propensity-matched GI bleeding-specific cohort

Abbreviations: DAPT, dual antiplatelet therapy; DOAC, direct oral anticoagulant; GI, gastrointestinal; LAAO, left atrial appendage occlusion; OAC, oral anticoagulation; SAPT, single antiplatelet therapy.

Note: Reporting of gastrointestinal bleeding source, endoscopic findings, timing of bleeding episodes, and therapy modification was heterogeneous across the included studies. Variables are presented according to the original study reports.

Supplementary Table S4. JBI critical appraisal of included cohort studies

Study	Q1	Q2	Q3	Q4	Q5	Q6	Q7	Q8	Q9	Q10	Q11
Lempereur et al. [14]	Y	Y	Y	Y	U	Y	Y	Y	U	U	Y
Kikuchi et al. [16]	Y	Y	Y	Y	U	Y	Y	Y	U	U	Y
Gallo et al. LOGIC [15]	Y	Y	Y	Y	Y	Y	Y	Y	U	U	Y
Darden et al. [17]	Y	Y	Y	Y	Y	Y	Y	Y	Y	Y	Y
Preisendörfer et al. [27]	Y	Y	Y	Y	Y	Y	Y	Y	Y	Y	Y

Abbreviations: JBI, Joanna Briggs Institute; Y, Yes; N, No; U, Unclear; NA, Not applicable.

Note: Q1–Q11 correspond to the 11 items of the Joanna Briggs Institute Critical Appraisal Checklist for Cohort Studies. Individual items were assessed as Yes, No, Unclear, or Not applicable, as appropriate. No numerical summary score or standardized overall risk-of-bias category was applied.

Supplementary Table S5. Principal sources of clinical and methodological heterogeneity precluding quantitative synthesis

Domain of heterogeneity	Variation across studies	Implication for quantitative synthesis
GI bleeding population	Dedicated GI bleeding cohorts versus separately reported GI bleeding subgroups	Limited comparability of study populations, baseline risk and GI-specific denominators
Definition of prior GI bleeding	Major GI bleeding, previous GI bleeding, registry-defined bleeding history or incompletely specified definitions	Inconsistent eligibility thresholds and baseline risk
Bleeding etiology and source control	Variable or incomplete reporting of angiodysplasia, ulcer disease, portal hypertensive bleeding, obscure bleeding and treatment success	Inability to stratify outcomes by bleeding phenotype, treatability or reversibility
Postprocedural antithrombotic therapy	DAPT, SAPT, anticoagulation-based regimens and individualized or incompletely reported strategies	No sufficiently uniform treatment exposure for pooled comparison
LAAO devices	WATCHMAN, WATCHMAN FLX, Amplatzer-based devices and mixed-device cohorts	Additional clinical heterogeneity related to device type and implantation protocols
Follow-up duration	Early postprocedural assessment to long-term follow-up exceeding two years	Different periods of exposure to recurrent bleeding and thromboembolic events
Outcome definitions	Variable definitions of recurrent GI bleeding, major bleeding, stroke/TIA, systemic embolism, DRT and mortality	Lack of harmonized outcome measures
Reporting metrics	Event counts, percentages, annualized rates, person-year rates and adjusted hazard estimates	Incompatible statistical measures
Study design	Retrospective cohorts, registries, subgroup analyses and propensity-matched observational data	Differences in selection, confounding control and analytical structure
Comparator structure	No comparator, GI versus non-GI groups or LAAO versus alternative management	No common comparison suitable for pooling

Abbreviations: DAPT, dual antiplatelet therapy; DRT, device-related thrombosis; GI, gastrointestinal; LAAO, left atrial appendage occlusion; SAPT, single antiplatelet therapy; TIA, transient ischemic attack.
